# Multi-Scale Modeling for Plasma-Enhanced Ammonia Decomposition over Carbides and Nitrides

**DOI:** 10.1021/acscatal.5c07452

**Published:** 2025-12-19

**Authors:** Saleh Ahmat Ibrahim, Qiang Li, Fanglin Che

**Affiliations:** † Department of Chemical Engineering, 542588 Worcester Polytechnic Institute, Worcester 01609, United States; ‡ Department of Chemical Engineering, 14710 University of Massachusetts Lowell, Lowell 01854, United States

**Keywords:** Ammonia decomposition, transition metal carbides and nitrides, zero-dimension plasma kinetics, multiscale simulation, non-thermal plasma, hydrogen production

## Abstract

Ammonia is a carbon-free hydrogen carrier, but its decomposition typically requires high temperatures over costly Ru-based catalysts due to the large barrier for N≡N bond formation. We develop a multiscale framework combining density functional theory, zero-dimensional plasma kinetics, and microkinetic modeling to elucidate how non-thermal plasma (NTP) enables low-temperature NH_3_ decomposition over Co-based carbides and nitrides, benchmarked against Ru and Co. Under thermal conditions, all catalysts are limited by N≡N bond formation, with Co_3_C­(001) most active owing to its negatively charged surface, strong N* binding, and low activation barriers of N≡N bond formation. Plasma-induced vibrational excitation of NH_3_ and its reactive radicals promotes a radical-driven •NH_2_–N* coupling pathway that dominates on Co_3_C­(001) and Co_3_N­(001), shifting the rate-limiting step to NH_3_^(v_1_)^ dissociation, increasing turnover frequencies by up to 6 orders of magnitude, and reducing the temperature needed to reach a turnover frequency of 5 s^–1^ from >680 °C (Ru and Co under thermal condition) to 267 °C (Co_3_C) and 415 °C (Co_3_N). These results identify Co-based carbides and nitrides as promising plasma-active catalysts for energy-efficient hydrogen production from ammonia.

## Graphical abstract

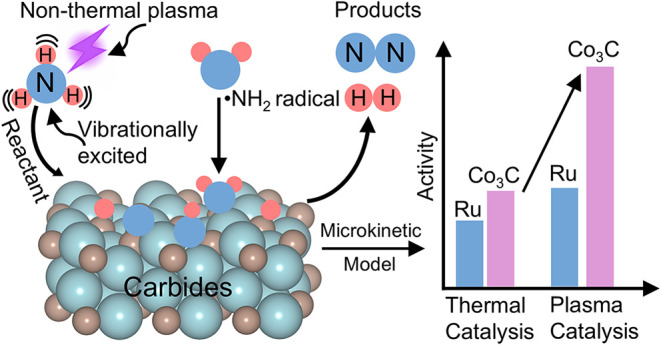

## 1 Introduction

Scaling energy-efficient hydrogen (H_2_) production is vital to decarbonizing the global energy system. However, H_2_’s low volumetric energy density (∼10 kJ/L at STP)^1^ requires energy-intensive liquefaction (−253 °C at 1 atm)^2,3^ for practical storage and transport, limiting its deployment as a widespread fuel. Ammonia (NH_3_) is a hydrogen-rich,^1^ carbon-free carrier with a high gravimetric energy density (142 kJ/g)^4^ and well-established global transportation infrastructure,^5^ making it a promising hydrogen carrier for distributed, sustainable chemical-to-energy conversion. Current hydrogen production from NH_3_ primarily relies on thermal-catalytic pathways, with photocatalytic and electrocatalytic decomposition emerging as alternatives.^6–8^ Although thermal-catalytic cracking is technologically mature, it is typically centralized and requires harsh reaction conditions above 550 °C,^9^ demanding high energy input^10^ that limits its feasibility for decentralized, on-demand applications. The high energy demand arises from the substantial barrier for N–H bond cleavage and N≡N bond formation, which often necessitates costly catalysts such as Ru.^11–13^

To reduce cost and improve practicality, researchers have turned to catalysts based on earth-abundant transition metals. Among these, transition-metal carbides (TMCs) and nitrides (TMNs) have emerged as promising alternatives due to their affordability and catalytic activity.^14–16^ In our recent multiscale simulation study on thermal NH_3_ cracking, Co_3_C demonstrated superior performance, achieving turnover frequencies (TOFs) approximately 10^4^ times higher than Co_3_N and 10^3^ times higher than Ru at 400 °C. This enhancement was attributed to the negatively charged Co_3_C surface, which attracts positively charged N* and strengthens the N* binding energy, thereby facilitating the rate-limiting step (N≡N bond formation) and reducing its activation barrier compared to Co_3_N and Ru.

Beyond replacing Ru with TMCs and TMNs, further improvements in low-temperature NH_3_ decomposition can be achieved by modifying the reaction environment through electrocatalysis, photocatalysis, or plasma catalysis. Electrocatalytic approaches can operate near room temperature but generally require large overpotentials (>0.6 V_RHE_)^17^ that can lead to poisoning of Pt-based anodes by adsorbed nitrogen (N*) and oxygenated nitrogen species.^18^ These side reactions also promote NO_*x*_ formation, reducing selectivity and overall energy efficiency. Photocatalytic systems such as Pt/TiO_2_ offer mild operating conditions but display intrinsically low activity.^19^ This limitation stems from poor light absorption, inefficient charge generation and separation, and slow multielectron kinetics, leading to charge accumulation, carrier recombination, and photocorrosion.^20,21^

Non-thermal plasma (NTP) catalysis provides a compelling route to overcome these challenges by enabling NH_3_ activation under ambient conditions through high-energy electrons (1–10 eV).^22–24^ Unlike conventional catalysis, where N–H bond cleavage and N≡N bond formation occur predominantly through surface-mediated pathways, NTP introduces additional reaction channels involving plasma-induced vibrationally excited NH_3_^(*v*)^ species and reactive radicals (such as •NH_2_, •NH, •N, and •H). These plasma-induced species can participate in both Langmuir–Hinshelwood (L-H) surface reactions and Eley–Rideal (E-R) radical-surface interactions, thereby reshaping the dominant reaction pathways, effectively bypassing the kinetic limitations of N–H bond cleavage and N≡N bond formation, shifting the optimal catalyst landscape from costly Ru-based systems to earth-abundant, cost-effective Co-based catalysts.^23,25,26^ In our previous work regarding plasma-enhanced NH_3_ decomposition over transition metals, we demonstrated that plasma-induced vibrational excitation and reactive radical formation shift the optimum nitrogen adsorption energy (*E*_N_) from −0.90 eV over Ru under thermal conditions to a weaker *E*_N_ of −0.51 eV under plasma conditions. This shift favors Co and several earth-abundant alloys (such as Ni_3_Mo, Fe_3_Cu, Ni_7_Cu, and Fe_15_Ni) as highly effective plasma catalysts.^27^

Based on our previous findings^27^ and reported literature,^25,28–30^ the plasma-catalyst interaction effects on catalytic NH_3_ decomposition are mainly via the following steps: (i) vibrational excitation of NH_3_ molecules, (ii) L-H catalytic surface reactions, and (iii) E-R radical-surface species interactions. The population of plasma-induced vibrationally excited species and reactive radicals is governed by vibrational temperature (T_v_), gas temperature (*T*_gas_), reduced electric field (E/N), and initial electron density (*n*_e_). Catalytic performance is further governed by catalyst properties, reaction temperature, and the partial pressures (*P*_i_) of reactants, products, and plasma-generated species.^3,31^

Despite these advances, the mechanistic nature of plasma-catalyst interactions on TMC and TMN materials remains poorly understood. A systematic framework (Scheme 1) that couples plasma kinetics with catalyst surface chemistry is therefore required to reveal how plasma-generated excited species (such as vibrationally excited molecules and reactive radicals) influence reaction mechanisms, shift rate-limiting steps (RLS), and ultimately determine catalytic performance over these emerging TMCs and TMNs catalysts.

**Scheme 1:**
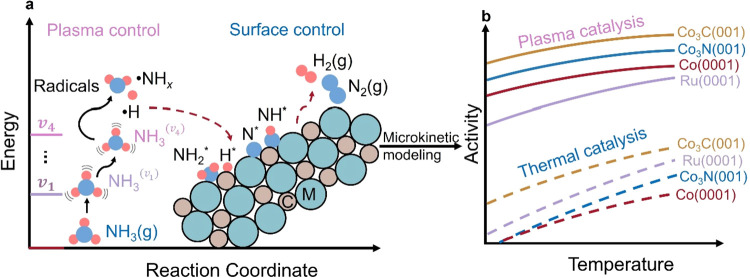
Plasma-Catalyst Interactions Predicted by Multi-Scale Simulations^a^

a (a) Scheme of plasma-catalyst interactions on atomic-scale energetics via density functional theory (DFT) calculations, including plasma-induced vibrational excitation, radical adsorption and radical-surface species interaction via E-R mechanism, surface reactions via L-H mechanism, reactant adsorption, and product desorption. (b) Microkinetic model (MKM)-predicted reaction activity (such as turnover frequency (TOF)) under both thermal and plasma conditions. The DFT energetics of the above interactions were incorporated as input data for establishing MKM.

Motivated by this knowledge gap, the present work integrates NTPs with Co-based carbides and nitrides to investigate low-temperature NH_3_ decomposition into H_2_. We focus on the most favorable catalytic facets, Co_3_C­(001) and Co_3_N­(001), and develop a comprehensive multiscale framework that combines density functional theory (DFT), plasma kinetics, and microkinetic modeling (MKM). This approach enables quantitative evaluation of vibrational excitation, radical-surface interactions, and surface–surface species reactions over Co_3_C­(001) and Co_3_N­(001) under plasma conditions, benchmarked against the conventional costly Ru(0001) and the plasma-active Co(0001) catalysts.

## 2 Methodology

### 2.1 Density Functional Theory (DFT) Calculation Details

The energetics of NH_3_ decomposition, including adsorption energies, reaction energies, and activation barriers, were systematically investigated on Co_3_C­(001), Co_3_N­(001), Ru(0001), and Co(0001) surfaces using plane-wave DFT. All calculations were performed with the Vienna *Ab initio* Simulation Package (VASP),^32^ employing the Perdew–Burke–Ernzerhof (PBE) exchange-correlation functional within the generalized gradient approximation (GGA)^33,34^ and projector-augmented wave (PAW) pseudopotentials.^35^ Dispersion interactions were accounted for through van der Waals corrections,^36^ and spin polarization^37^ was included to accurately describe the magnetic properties of the Co-based systems. The optimized lattice constants for Co_3_C, Co_3_N, Ru, and Co were 4.393 Å, 4.540 Å, 3.790 Å, and 2.505 Å, respectively, which are in close agreement with experimental and previously reported theoretical values (Figure S1 and Table S1).^38–40^ Convergence criteria were set to ensure robust accuracy, with total energy and force thresholds of 10^–5^ eV and 0.02 eV Å^–1^.^41^

We selected Co_3_C and Co_3_N as representative Co-based carbide and nitride catalysts because their formation enthalpies are lower than those of other phases (e.g., Co_2_C and Co_2_N/Co_4_N), making them the most thermodynamically stable bulk structures for constructing reliable surface models (Figure S2). Previous experimental and computational studies report that Co_3_C exhibits higher kinetic and thermal stability than Co_2_C, and that Co_3_N is the phase most frequently synthesized and employed in catalysis and energy applications, supporting our finding that these phases are robust under reaction conditions (i.e., temperature range of 300–800 K and pressures spanning 0–50 GPa).^42–47^ In addition, we performed first-principles phase-diagram calculations and showed that Co_3_C and Co_3_N maintain positive Gibbs free energies of decomposition across the relevant hydrogen pressures (thermal condition: 10^–4^ to 0.25 bar; plasma condition: 0.25 to 0.6 bar) and temperatures (400 to 800 °C). Our results demonstrate that both phases remain thermodynamically stable under the studied thermal and plasma-assisted NH_3_-decomposition conditions (Figure S3). More details regarding the selection and stability of carbide and nitride phases are shown in Section 2 of Supporting Information (SI).

Surface models were constructed using *p*(2 × 2) periodic slabs separated by a 20 Å vacuum layer to avoid interactions between periodic images. The bottom three atomic layers were fixed during structural relaxation, while the remaining layers and all adsorbates were fully relaxed. The Brillouin zone was sampled with a (3 × 3 × 1) Monkhorst–Pack k-point mesh. The most stable surface terminations and adsorption configurations were identified through comparison of surface energies and adsorption energies, as detailed in the Supporting Information. The possible adsorption sites for NH_3_ and its intermediates on each surface are illustrated in Figure S4. Transition states^48^ for elementary reaction steps were located using the climbing image nudged elastic band (CINEB) method^49^ and further refined using the improved dimer method.^50^ Each transition state was confirmed by the presence of a single imaginary frequency.^51^ The resulting data set of reaction energies, activation barriers, and model parameters serve the primary input for the microkinetic model (Tables S2–S5). Adsorption configurations (Figures S5–S14) are provided in Section 3 of the SI.

### 2.2 Zero-Dimensional Plasma Kinetic Model (ZDPlasKin)

Plasma-phase chemistry was modeled using a zero-dimensional (0D) kinetic framework implemented in ZDPlasKin^52^ and coupled with BOLSIG+ to solve the steady-state Boltzmann equation for the electron energy distribution function (EEDF).^53^ This approach enables accurate estimation of electron-impact reaction rates and energy transfer processes under nonequilibrium dielectric-barrier discharge (DBD) conditions. The model tracks the time-dependent evolution of plasma species densities through a system of coupled rate equations, incorporating electron-impact excitation, ionization, dissociation, recombination, and vibrational–translational (V-T) coupling processes.

The ZDPlasKin simulations were carried out using an experimentally measured reduced electric field of 126 Td.^27^ The gas density of 1.091 × 10^19^ cm^–3^ was obtained from the ideal-gas relation at 673 K and 1 bar, and the system’s residence time was calculated to be 0.1814 s based on the experimental reactor geometry and gas flow rate.^27^ A surface site density of 1.55 × 10^15^ cm^–2^ was used to represent the available adsorption capacity of a catalytic surface, providing a physically meaningful basis for subsequent integration with surface reaction models. With an initial electron density of 1.25 × 10^15^ cm^–3^, and an electron temperature of 1.41 eV, the model reproduces the experimentally observed plasma-only NH_3_ conversion (2.7%). The resulting concentrations of gas-phase molecules (NH_3_, N_2_, H_2_), vibrationally excited NH_3_ species (NH_3_^*v*^) and reactive radicals (•NH_2_, •NH, •H, and •N) under plasma-only conditions are shown in Figure S15. These plasma-generated species densities serve as boundary conditions for microkinetic simulations, enabling self-consistent treatment of plasma-catalyst interactions.

### 2.3 Microkinetic Modeling (MKM)

The kinetics of NH_3_ decomposition on Co_3_C­(001), Co_3_N­(001), Ru(0001), and Co(0001) surfaces under thermal and NPT conditions were further quantified using a mean-field microkinetic modeling (MKM)^54,55^ framework based on transition state theory^48^ (TST). Thermodynamic and kinetic parameters, derived from DFT energetics and vibrational partition functions, were processed using the Python Multiscale Thermochemistry Toolbox (pMuTT)^56^ under harmonic oscillator approximation.^57^ These data were used to calculate the forward reaction rate constants and equilibrium constants. Reverse reaction rate constants were calculated from the forward rates and equilibrium constants.^58,59^ Adsorption rates were derived from collision theory.^60^ For adsorption steps, we used an initial sticking coefficient of 0.5 for NH_3_, H_2_, and N_2_, consistent with prior studies.^6,61^

The reaction network was solved in a plug-flow reactor (PFR),^62^ coded as continuous stirred-tank reactor in Cantera.^63^ The reactor was assumed to be isothermal, with negligible pressure drop and no axial dispersion. Reactor dimensions and conditions were explicitly defined to ensure reproducibility: an initial length of 10 cm (optimized to achieve 1% NH_3_ conversion under each condition), a feed flow rate of 35 cm^3^/min, and a porosity of 0.95, operated at 400 °C and 1 atm. The superficial gas velocity was calculated accordingly. The geometric cross-sectional area was 1 cm^2^, and the catalyst surface area per unit reactor volume was set to 3000 cm^–1^. These parameters define the total exposed catalyst surface area per segment, which determines the overall reaction rate. The evolution of surface coverages along the reactor length was described by coupled ordinary differential equations (ODEs), accounting for net production and consumption from the proposed reaction networks (Scheme 1). After establishing MKM, the sensitivity analysis, so-called the degree of rate control (DRC),^64,65^ was then carried out to identify rate-limiting steps under both thermal and NTP conditions.

To assess the influence of reverse chemistry, we expanded the reaction network to include all ammonia-synthesis steps, the reverse pathway of ammonia decomposition (Table S6), under plasma conditions, including plasma-induced N_2_ vibrational excitation. As shown in Figure S16, incorporation of these reverse pathways has a negligible effect on NH_3_ decomposition rates for Co_3_C, Co_3_N, Ru, and Co. TOFs from the original and extended MKM models are nearly identical. Consistent with our previous findings^27^ that NH_3_ synthesis remains kinetically insignificant under the conditions considered. Therefore, subsequent discussion focuses exclusively on the NH_3_ decomposition pathway. Additional MKM implementation details are provided in Section 5 of the SI.

## 3 Results and Discussion

### 3.1 Kinetic Properties of Thermal NH_3_ Decomposition

Thermal NH_3_ decomposition over transition metal carbides (TMCs) and nitrides (TMNs) proceeds through stepwise sequence of ammonia adsorption and surface dehydrogenation (NH_3_* ⇌ NH_2_* ⇌ NH* ⇌ N*), followed by the recombination of adsorbed N* and H* to form N_2_* and H_2_*, and subsequent product desorption. To quantify the reaction kinetics, we developed a MKM based on this reaction network. The model was evaluated using pure NH_3_ at 1 atm, with temperatures ranging from 400 to 800 °C to represent typical industrial conditions for NH_3_ decomposition.^66,67^ Since the turnover frequency (TOF) predicted by the microkinetic model for NH_3_ decomposition is highly sensitive to the ammonia conversion assumed in the simulation, we fixed NH_3_ conversion at 1% to enable consistent comparison of catalytic activity and surface coverages across examined catalysts.

The MKM results show that Co_3_C­(001) is the most active catalyst (Figure 1a). At 400 °C, Co_3_C­(001) achieves a TOF nearly 4 orders of magnitude higher than Co_3_N­(001), Ru(0001), and Co(0001). Across the entire temperature range, Co_3_C­(001) consistently maintains higher TOFs than Co_3_N­(001), Ru(0001), and Co(0001). Additionally, we performed a degree of rate control (DRC) analysis^64^ to identify the rate-limiting step (RLS). Our DRC analysis demonstrates that N≡N coupling step (2N* ⇌ N_2_* + *) is the RLS across all four catalyst surfaces (Figure 1b–d). On Co_3_C­(001), NH_2_* dehydrogenation (NH_2_* + * ⇌ NH* + H*) emerges as the secondary RLS, while on Co_3_N­(001), NH* dehydrogenation (NH* + * ⇌ N* + H*) becomes the secondary RLS.

**Figure 1:**
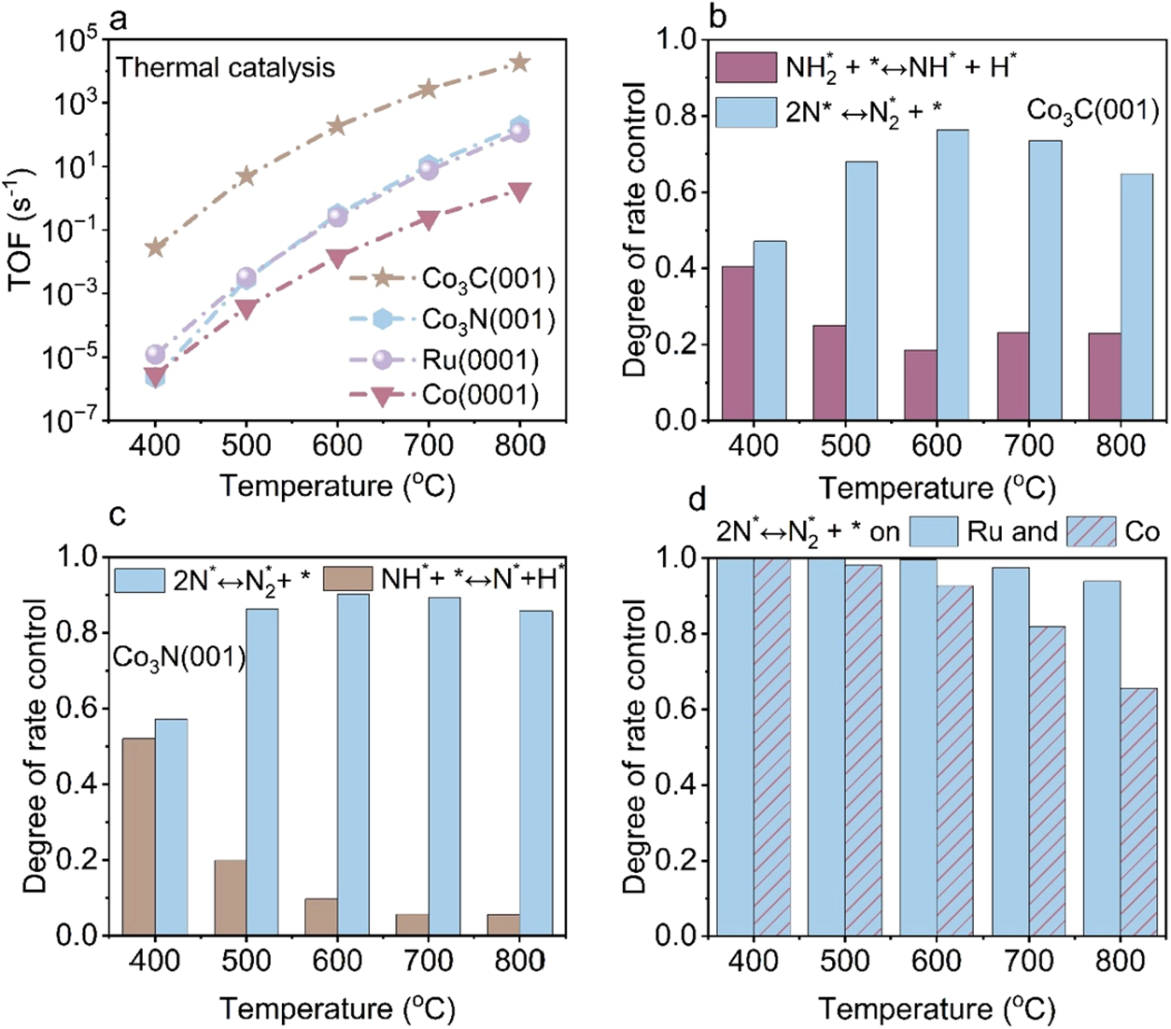
Microkinetic modeling of thermal NH_3_ decomposition over Co_3_C­(001), Co_3_N­(001), Ru(0001), and Co(0001) surfaces. (a) Temperature-dependent TOFs showing that Co_3_C­(001) exhibits substantially higher activity than Co_3_N­(001), Ru(0001), and Co(0001). DRC analysis identifying the rate-limiting steps (RLSs) for (b) Co_3_C­(001), (c) Co_3_N­(001), and (d) Ru(0001) and Co(0001). Solid bars correspond to Ru(0001), while hatched bars indicate Co(0001) in panel (d). N≡N bond formation (2N* ⇌ N_2_* + *) emerges as the primary RLS across all examined catalysts.

A notable feature on Co_3_C­(001) is the nonmonotonic, U-shaped temperature dependence of the DRC values for both N≡N coupling and NH_2_* dehydrogenation (Figure 1b). This behavior reflects temperature-driven changes in surface species coverages and their relative kinetic influence. Across the investigated temperature range (400 to 800 °C), N* remains the most abundant surface species on Co_3_C­(001), consistent with the high activation free energy of the N≡N bond formation step (Figure S17a). As temperature increases from 400 to 600 °C, the coverage of N* rises from 0.46 to 0.76 ML, corresponding to the increasing DRC value associated with N≡N bond formation. At higher temperatures (600 to 800 °C), the coverage of N* decreases from 0.76 to 0.5 ML, which corresponds to the decreased DRC value for N≡N bond formation (Figure S17b).

For the secondary rate-limiting step, NH_2_* dehydrogenation, the DRC decreases from 0.40 at 400 °C to 0.18 at 600 °C as NH_3_* and NH_2_* surface coverages decrease. Between 600 and 800 °C, the coverages of NH_3_* and NH_2_* remain nearly constant; however, the increasing Gibbs free energy barrier for NH_2_* dehydrogenation leads to a slight upward shift in its DRC value (Figure S17).

The high performance of Co_3_C­(001) under thermal conditions can be directly attributed to its negatively charged surface. Bader charge analysis shows that Co_3_C surface is negatively charged (−0.04 e/Å^2^), whereas Co_3_N surface is positively charged (+0.03 e/Å^2^). Metallic Co surface is charge-neutral (0.0 e/Å^2^). The electron-rich Co_3_C surface binds key intermediate N* most strongly (*E*_N_ = −0.62 eV), metallic Co surface shows intermediate binding (*E*_N_ = −0.44 eV), and the electron-poor Co_3_N surface physically binds N* (*E*_N_ = 0.01 eV) (Figure S18a). The binding strength of the key intermediate N* linearly correlates to the activation barriers of RLS, N≡N bond formation (Figure S18b). Thus, negatively charged Co_3_C­(001) surface with the strongest *E*_N_, exhibits the lowest N≡N bond formation barrier (1.82 eV) and thus, the highest observed catalytic activity.

### 3.2 NH_3_ Gas Phase Activation under Plasma via ZDPlasKin

Although the thermal catalysis results clarify the underlying decomposition pathways of NH_3_ over Co_3_C­(001), Co_3_N­(001), Ru(0001), and Co(0001), the high temperatures required for these processes motivate the exploration of nonthermal plasma (NTP) approaches. Plasma-assisted NH_3_ decomposition can activate NH_3_ under significantly milder conditions, alter the reaction mechanism, and shift the RLS, thereby lowering the temperature requirement and reducing dependence on scarce and costly noble metals such as Ru that dominate conventional high-temperature catalysis.^27^ In NTP environments, a complex mixture of reactive species, including vibrationally excited NH_3_ molecules and reactive radicals, is generated through hot electron-driven processes.^68,69^ However, the specific roles and relative contributions of these plasma-induced species to NH_3_ decomposition over TMCs and TMNs remain poorly understood. To address this gap, we extended our microkinetic modeling framework to incorporate two plasma-enabled activation channels: (i) vibrational excitation only, and (ii) vibrational excitation coupled with reactive radical-surface (E–R) interactions.

To accurately evaluate these plasma effects within the MKM, it is essential to first quantify the concentrations of vibrationally excited species and reactive radicals generated in the plasma-only phase. To address this, we employed a zero-dimensional plasma kinetic solver (ZDPlasKin) to simulate the temporal evolution of species densities within the plasma reactor. ZDPlasKin integrates a comprehensive database of elementary plasma processes with a system of coupled differential equations to describe species dynamics under discharge conditions.

The ZDPlasKin simulations^24,52,70^ reveal that NH_3_ activation proceeds primarily through vibrational excitation, followed by the formation of reactive radicals (Figure S15). These computed concentrations of NH_3_^*v*^, •NH_2_, •NH, •N, and •H were subsequently incorporated into the microkinetic model as boundary conditions, enabling us to quantify their direct impact on surface reaction kinetics and identify how plasma modifies decomposition pathways over TMC and TMN catalysts.

### 3.3 Plasma-Induced Vibrational Excitation-Enhanced NH_3_ Decomposition

To isolate the effect of vibrational excitation and radical-surface interactions induced by plasma, we first integrated four vibrational states of NH_3_ into the MKM, building directly on the thermal framework. The vibrational excitation of NH_3_^*v*^ species enhances the TOF by approximately one to 2 orders of magnitude relative to thermal conditions, rising from 2.80 × 10^–2^ s^–1^ to 3.15 × 10^–1^ s^–1^ on Co_3_C­(001), from 8.12 × 10^–7^ s^–1^ to 4.40 × 10^–6^ s^–1^ on Co_3_N­(001), and from 2.83 × 10^–6^ s^–1^ to 4.91 × 10^–3^ s^–1^ on Co(0001) at 400 °C. In contrast, the TOF on Ru(0001) is nearly the same upon vibrational excitation, varying slightly from 1.19 × 10^–5^ to 1.20 × 10^–5^ s^–1^ at 400 °C (Figure 2a).

**Figure 2:**
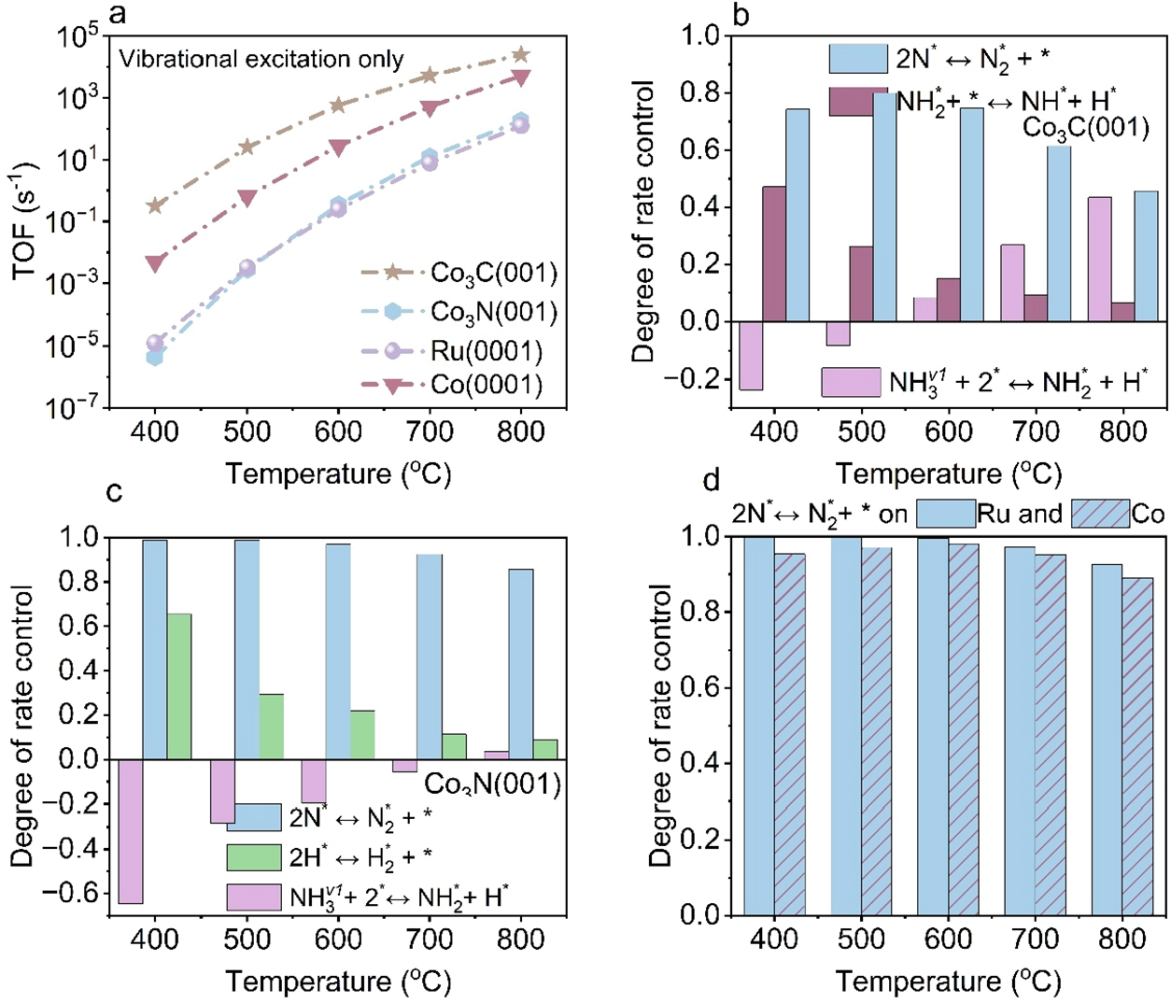
Microkinetic modeling of predicting the TOFs and DRC analysis for plasma-assisted NH_3_ decomposition via vibrational excitation only. The temperature-dependent (a) TOFs and DRC analysis over (b) Co_3_C­(001), (c) Co_3_N­(001), (d) Ru(0001) and Co(0001).

To gain deeper insight into the mechanistic origins of such TOF enhancement, we performed a DRC analysis to identify how vibrational excitation alters the RLSs and how the evolving activation energetics and surface coverages collectively reshape the overall reaction kinetics. The DRC analysis of NH_3_ decomposition under plasma-induced vibrational excitation identifies that N≡N bond formation remains the primary RLS across all examined catalysts and temperatures, consistent with the behavior observed in thermal catalysis (Figures 1 and 2). On Co_3_C­(001), NH_2_* dehydrogenation persists as the secondary RLS (Figure 2b), whereas on Co_3_N­(001), the secondary RLS shifts from NH* dehydrogenation to the H–H recombination step (Figure 2c). In contrast, N≡N bond formation remains as the only RLS on Ru(0001) and Co(0001), consistent with thermal catalysis behavior (Figure 2d). Additionally, the dissociative adsorption of first-level vibrationally excited NH_3_ (NH_3_^(v_1_)^ + * ⇌ NH_2_* + H*) over Co_3_C­(001) and Co_3_N­(001) exhibits negative DRC values at low temperatures, indicating an inhibitory effect on the overall reaction rate. As the temperature increases, however, this step transitions to a positive DRC contribution, emerging as the third RLS.

To elucidate the physical origin of these shifts in rate control and identify the underlying factors driving the temperature-dependent behavior, we analyzed the temperature dependence of the Gibbs activation barriers (Δ*G*_a_^‡^), surface coverages, and intrinsic reaction rates of the RLSs. On Co_3_C­(001), the results show that the Δ*G*_a_^‡^ for NH_3_^(v_1_)^ dissociative adsorption (Figure 3a) increases markedly with temperature, while those for NH_2_* dehydrogenation and N_2_* formation remain nearly constant. At low temperatures, the rapid NH_3_^(v_1_)^ dissociation produces abundant NH_2_* intermediate that accumulate on the surface (Figure 3b), confirming NH_2_* dehydrogenation as the secondary RLS and making NH_3_^(v_1_)^ dissociation an inhibition step on Co_3_C­(001). As the temperature increases, the NH_2_* coverage decreases and the fraction of empty sites grows, while the higher barrier for NH_3_^(v_1_)^ dissociation slows this process relative to NH_2_* dehydrogenation. Consequently, NH_3_^(v_1_)^ dissociation transitions from an inhibitory step to a secondary RLS (Figure 3c). Throughout the examined temperature range, N≡N bond formation exhibits the lowest reaction rate (Figure 3c), thereby dominating the overall kinetics and serving as the primary rate-limiting step for NH_3_ decomposition on Co_3_C­(001). In addition, the reaction rates of both primary and secondary RLSs, N≡N bond formation and NH_2_* dehydrogenation, exhibit accelerated reaction rates under plasma-induced vibrational excitation, ultimately resulting in enhanced TOFs over Co_3_C­(001) (Figure 3c).

**Figure 3:**
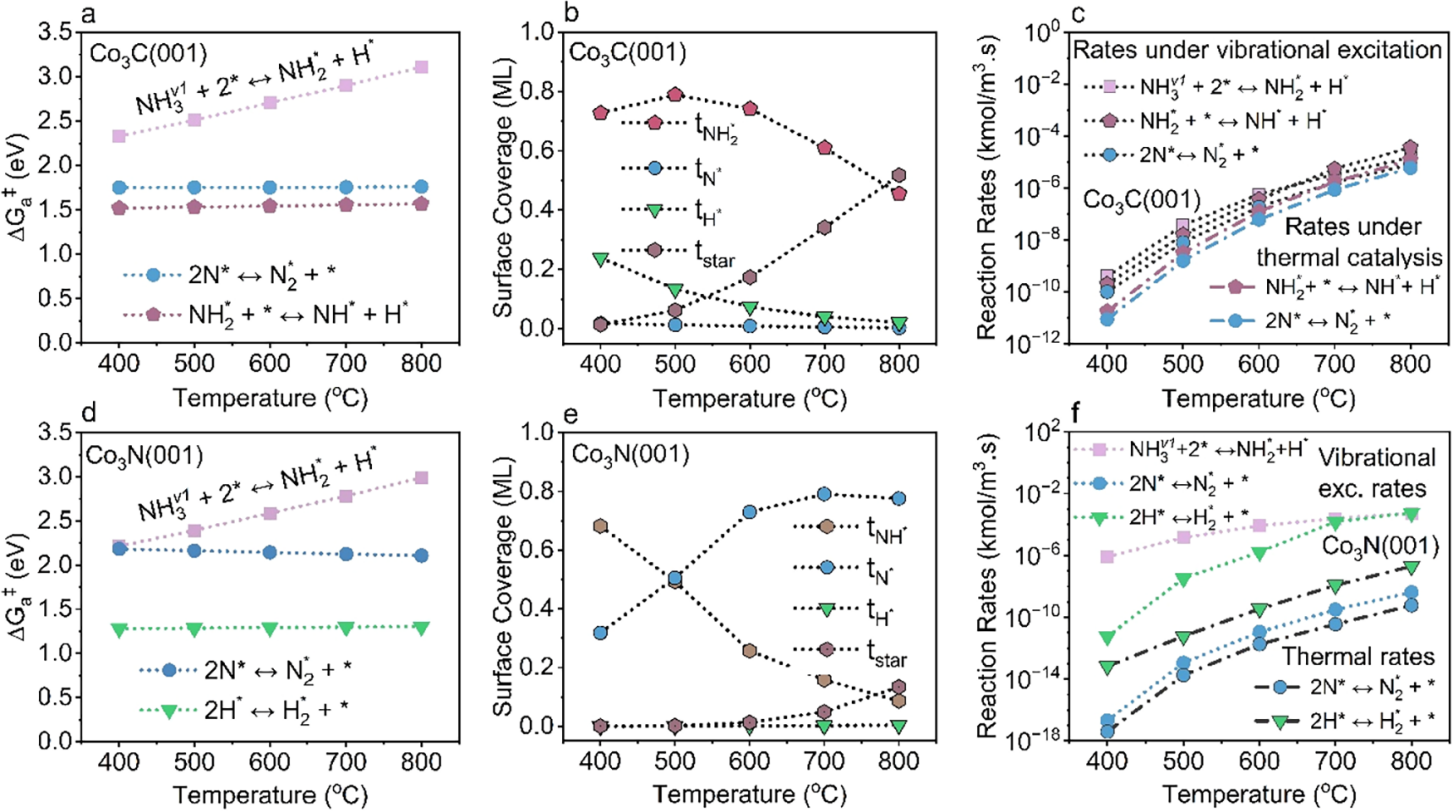
Mechanistic analysis of Co_3_C­(001) and Co_3_N­(001) under plasma-induced vibrational excitation. The temperature-dependent evolution of Gibbs activation barriers (Δ*G*_a_^‡^), surface coverages, and reaction rates for (a–c) Co_3_C­(001); (d–f) Co_3_N­(001).

For Co_3_N­(001), the NH_3_^(v_1_)^ dissociation at low temperatures (Figure 3d) generates an excess of NH* and N* intermediates that accumulate on the surface due to the high barrier of N≡N bond formation (Figure 3e). As a result, N≡N bond formation serves as the RLS, while the limited availability of vacant sites makes NH_3_^(v_1_)^ dissociation an inhibitory step. With increasing temperature, the fraction of empty sites grows, eliminating the inhibitory effect of NH_3_^(v_1_)^ dissociation (Figure 3e). Across the examined temperature range, N≡N and H–H bond formations exhibit the primary and secondary RLSs for NH_3_ decomposition on Co_3_N­(001), respectively (Figure 3f). Additionally, under plasma-induced vibrational excitation, the coverage of N* ranges from 0.18 monolayer (ML) (thermal condition) to 0.32 ML and the coverage of H* increases from 0.0015 ML (thermal conditions) to 0.019 ML. These changes enhance the N_2_* formation rate from 3.93 × 10^–20^ (thermal) to 2.10× 10^–17^ kmol m^–3^ s^–1^ and H_2_* formation rate from 6.86 × 10^–14^ (thermal) to 5.80 × 10^–12^ kmol m^–3^ s^–1^, leading to improved TOFs under plasma-induced vibrational excitation only condition.

On Co(0001), N≡N formation remains the primary rate-limiting step under vibrational-excitation-only conditions. Vibrational excitation of NH_3_ markedly accelerates this step, increasing the N≡N formation rate from 9.01 × 10^–19^ (thermal) to 1.50 × 10^–12^ kmol m^–3^ s^–1^ and thereby producing a substantial TOF enhancement under plasma-induced vibrational excitation (Figure S19). This acceleration arises because vibrationally excited NH_3_ lowers the activation barrier for N–H bond cleavage, which is particularly beneficial for Co(0001), a surface characterized by weaker N* binding and relatively high N–H dissociation barriers.^27^

On Ru(0001), N≡N formation also remains the primary rate-limiting step under vibrational-excitation-only conditions. Ru(0001) remains almost fully saturated with N* under both thermal and plasma-induced vibrational excitation only conditions, with its coverage slightly increasing from 0.9990 to 0.9997 ML. This change raises the N≡N formation rate from 3.812 × 10^–15^ (thermal) to 3.817 × 10^–15^ kmol m^–3^ s^–1^, resulting in a slight increase in TOF under plasma-induced vibrational excitation only conditions.

### 3.4 Reactive Radical-Enhanced NH_3_ Decomposition under Plasma Conditions

Nonthermal plasma generates a diverse array of reactive radicals, as revealed by the ZDPlasKin simulations, including •NH_2_, •NH, •N, and •H (Figure S15 **)**. To elucidate the contribution of these plasma-generated radicals to the enhanced TOFs observed in plasma-assisted NH_3_ decomposition, we incorporated radical-surface interactions into the MKM via the Eley–Rideal (E–R) mechanism.

The E–R mechanism is formulated based on several key assumptions that capture the essential physics of plasma-surface interactions^27^: (i) plasma-generated radicals can adsorb onto the catalyst surface, with adsorption governed by entropy losses;^71^ (ii) plasma-generated radicals can directly react with adsorbed surface species, producing new radicals, adsorbed intermediates, or gas-phase molecules; (iii) radicals can only abstract surface H* to form new radicals or gas-phase molecular species; (iv) N_2_H_*x*_ intermediates do not participate in radical-driven reactions leading to higher-order N_3_ species; and (v) all radical-surface species reactions proceeding via the E–R mechanism are considered enthalpically barrierless, with their kinetics determined solely by the entropy loss associated with radical adsorption.^31,72^

Based on these principles, 37 possible reactions were initially identified (Table S3). Using DFT-derived energetics, we performed a thermodynamic screening to assess the favorability of each pathway. The analysis showed that reactions forming new surface intermediates are generally more favorable than those generating additional radicals or gas-phase products. For example, the reaction •NH + NH_2_* ⇌ NH_2_–NH* is preferred over •NH + NH_2_* ⇌ •NH_2_ + NH*. Consequently, all energetically unfavorable steps yielding extra radicals or gaseous species were excluded from the reaction network. Furthermore, based on our extended MKM results, the steps leading to NH_3_ synthesis have no meaningful influence on the kinetics of NH_3_ decomposition (Figure S16). Thus, the three reactions associated with NH_3_ formation were also excluded. In cases where multiple pathways could produce the same surface intermediate, such as NH-N* formed via •NH + N* ⇌ NH-N* or •N + NH* ⇌ NH–N*, the thermodynamically favorable route was selected. After this systematic refinement, the radical-surface reaction network was reduced to 19 elementary steps (Table S4), which were subsequently integrated into the MKM.

Incorporating both vibrational excitation and radical-surface chemistry dramatically enhances NH_3_ decomposition across the examined catalysts. At 400 °C, the TOF on Co_3_C­(001) increases from 2.80 × 10^–2^ (thermal) to 63 s^–1^ (plasma) and on Co_3_N­(001) from 8.12 × 10^–7^ s^–1^ (thermal) to 2.64 s^–1^ (plasma) (Figure 4a,b). Co(0001) also shows substantial TOF enhancement, rising from 2.83 × 10^–6^ (thermal) to 6.54 × 10^–3^ s^–1^ (plasma) (Figure 4c). However, Ru(0001) shows only a slight improvement under plasma conditions (Figure 4d), with TOF varying only from 1.19 × 10^–5^ s^–1^ (thermal) to 1.21 × 10^–5^ s^–1^ at 400 °C. Among all catalysts examined, Co_3_C­(001) demonstrates the most pronounced enhancement under plasma conditions relative to thermal catalysis and remains the most active catalyst for NH_3_ decomposition in the plasma environment (Figure 5).

**Figure 4:**
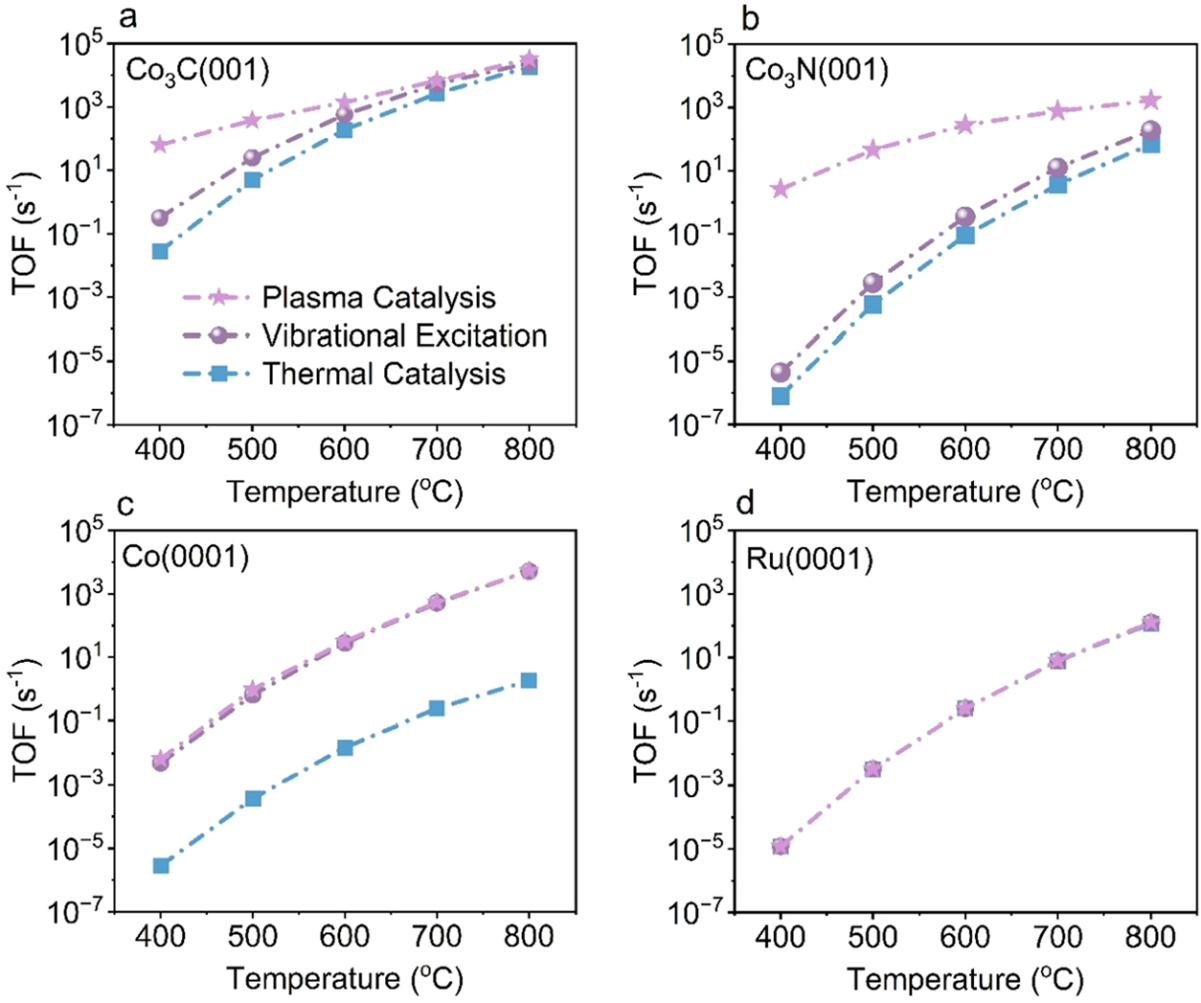
MKM-predicted TOFs of plasma-assisted NH_3_ decomposition, including vibrational excitation and radical-surface interactions. Temperature-dependent TOFs for (a) Co_3_C­(001), (b) Co_3_N­(001), (c) Co(0001), and (d) Ru(0001).

**Figure 5:**
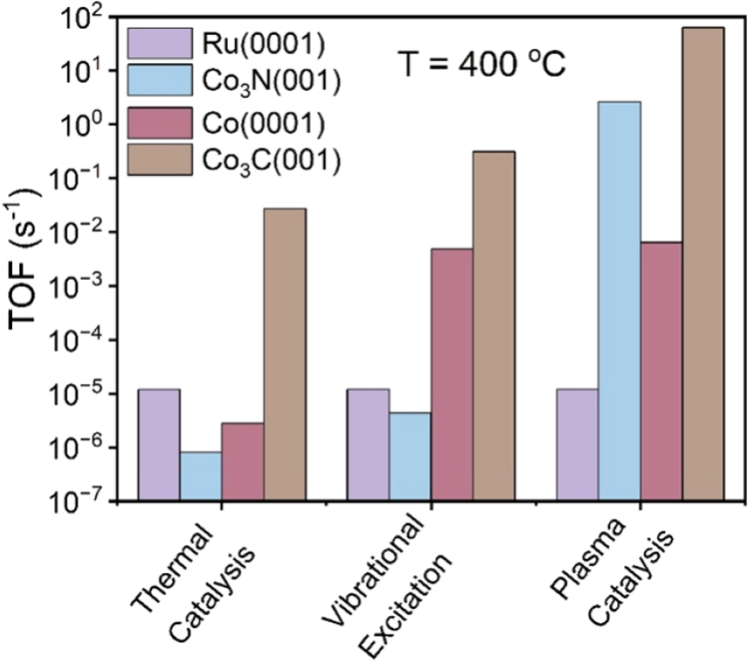
MKM-predicted TOFs of all three catalysts at 400 °C, highlighting the superior activity of Co_3_C­(001) under thermal and plasma conditions.

To uncover the mechanistic origin of these enhancements, we performed DRC analysis. The RLS on Co_3_C­(001) and Co_3_N­(001) shifts from N≡N bond formation under thermal and vibrational-excitation-only conditions, to the dissociative adsorption of first-level vibrationally excited NH_3_^(v_1_)^ across the examined temperature range (Figure 6a,b). On Co_3_C­(001), the NH_2_* dehydrogenation step remains the secondary RLS (Figure 6a), whereas on Co_3_N­(001), the H–H bond formation step still serves as the secondary RLS (Figure 6b). In contrast, for Ru(0001) and Co(0001), N≡N bond formation remains the only RLS under both thermal and plasma conditions (Figure 6c,d).

**Figure 6:**
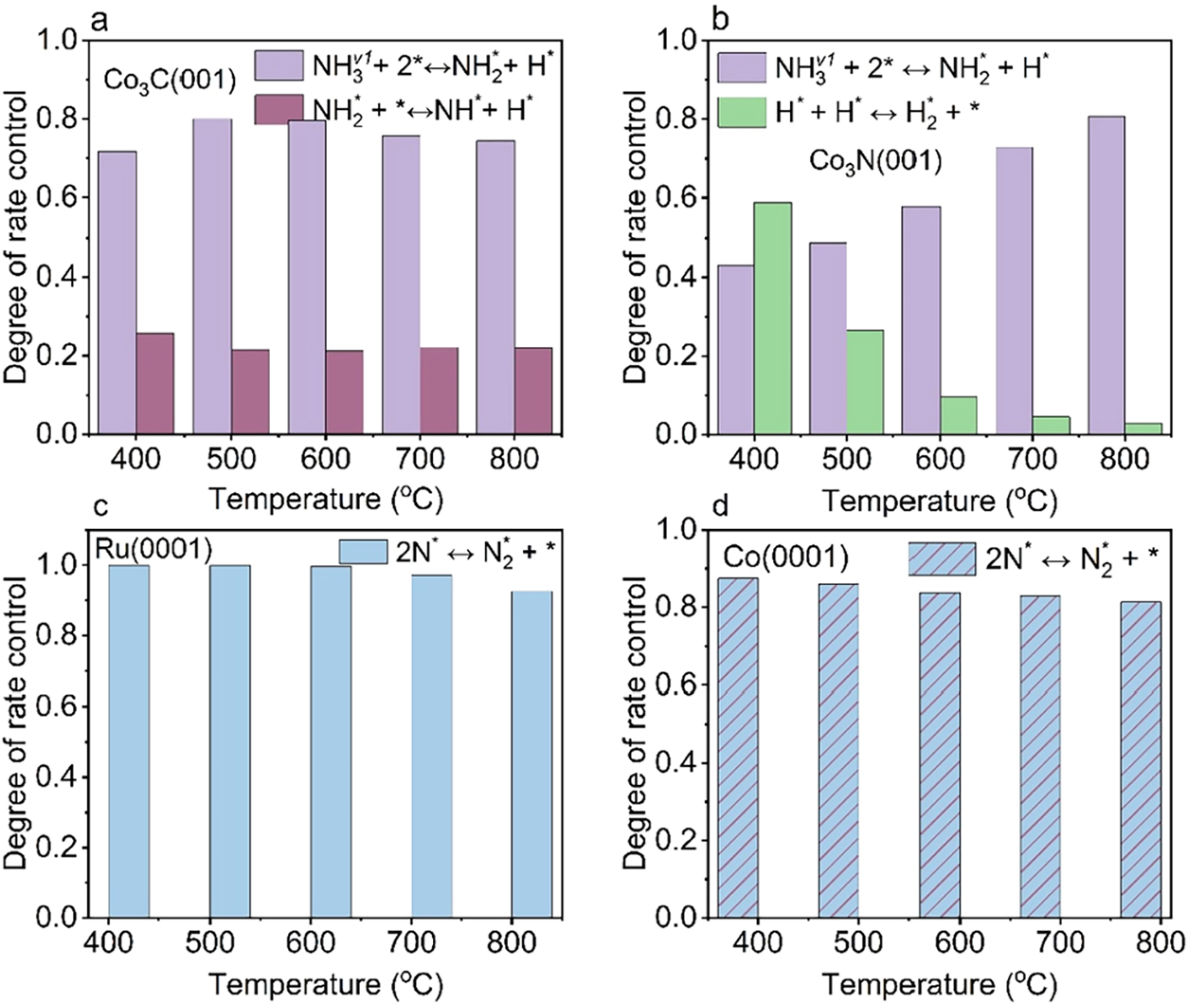
DRC analysis of NH_3_ decomposition as a function of temperature over (a) Co_3_C­(001), (b) Co_3_N­(001), (c) Ru(0001), and (d) Co(0001) under plasma conditions, including both vibrational excitation and radical-surface interactions via E-R mechanism.

To further understand the mechanistic origin of these RLS shifts and how plasma-induced radicals reshape the reaction kinetics, we further examined elementary reaction rates and Gibbs activation barriers (Δ*G*_a_^‡^ as a function of temperature for Co_3_C­(001) and Co_3_N­(001)). Under plasma conditions, the reaction mechanism is dominated by the E-R pathway, in which gas-phase •NH_2_ radicals react directly with surface N* species (•NH_2_ + N* ⇌ NH_2_–N*), followed by the dehydrogenation of NH_2_–N* to form N_2_* over both Co_3_C­(001) and Co_3_N­(001). This radical-driven pathway becomes the most favorable reaction pathway, with its reaction kinetics far exceeding that of the N≡N bond formation step proceeding via the L-H mechanism typically observed under thermal and vibrational-excitation-only conditions over Co_3_C­(001) and Co_3_N­(001) (Figure 7a,b).

**Figure 7:**
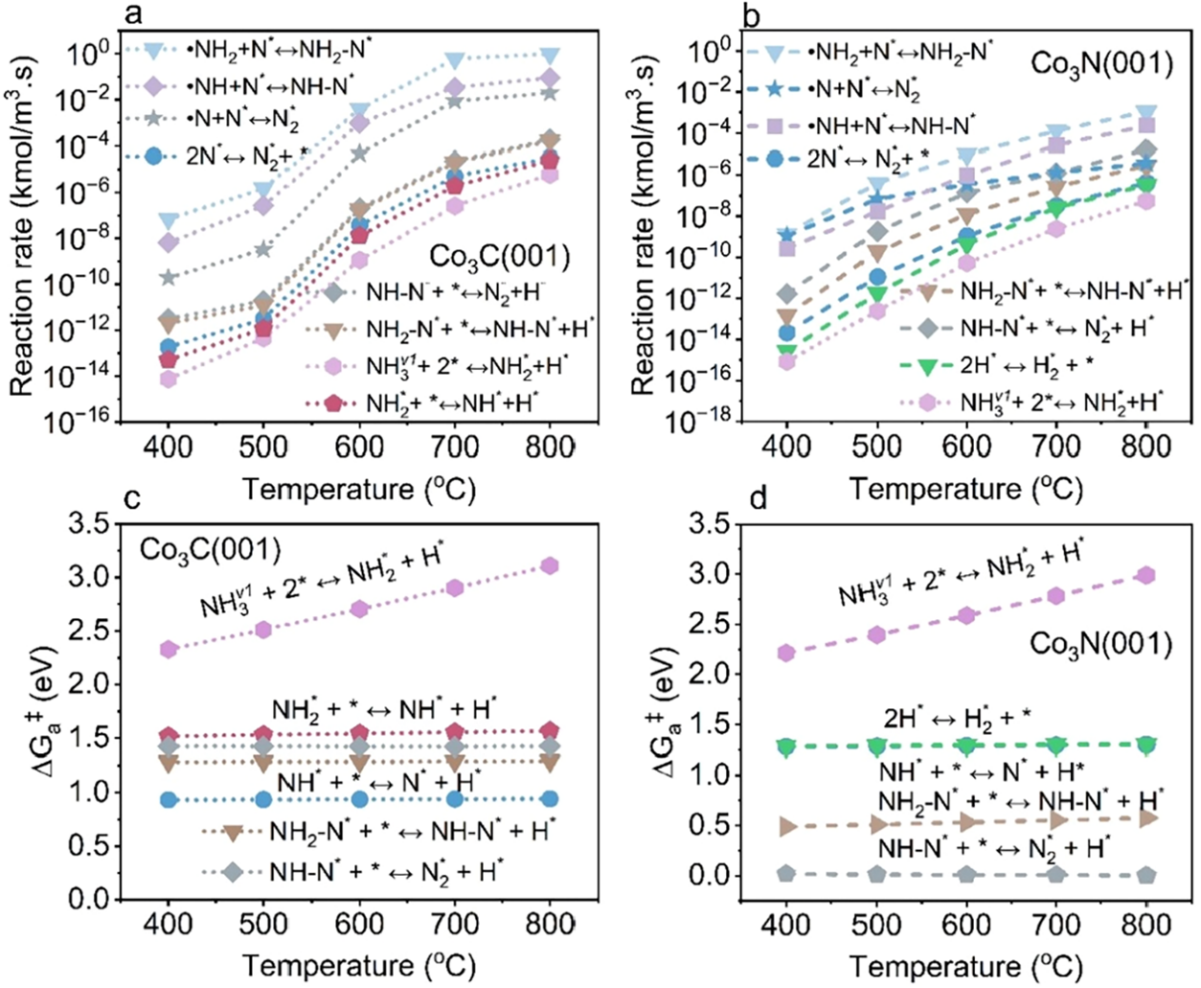
Analysis of elementary reaction rates and Gibbs activation barriers (Δ*G*_a_^‡^) under plasma conditions (including vibrational excitation and reactive radicals). (a, b) Reaction rates of key elementary steps, (c, d) Δ*G*_a_^‡^ of key elementary steps for Co_3_C­(001) and Co_3_N­(001), respectively.

On Co_3_C­(001) and Co_3_N­(001), the dissociative adsorption of first-level vibrationally excited NH_3_^(v_1_)^ emerges as the primary RLS due to their high activation barrier (Figure 7c,d). At 400 °C under plasma conditions, the corresponding RLS of NH_3_^(v_1_)^ dissociative adsorption rates are 7.62 × 10^–15^ and 8.42 × 10^–16^ kmol m^–3^ s^–1^, significantly exceeding N≡N bond formation rates (RLS under thermal) of 8.79 × 10^–18^ and 3.93 × 10^–20^ kmol m^–3^ s^–1^ on Co_3_C­(001) and Co_3_N­(001), respectively. These findings explain the dramatic enhancement in TOFs observed on Co_3_C­(001) and Co_3_N­(001) surfaces.

To further elucidate how the incorporation of C and N drive the shift in the rate-limiting step on Co_3_C­(001) and Co_3_N­(001), i.e., from N≡N bond formation on Co(0001) to dissociative adsorption of vibrationally excited NH_3_^(v_1_)^ under plasma conditions, we analyzed the surface coverages of key intermediates. On both Co_3_C­(001) and Co_3_N­(001), the N* coverage remains very low, below 0.1 monolayer (ML), whereas on Co(0001), N* nearly saturates the surface at close to 1 ML (Figure S20). The large difference in N* surface coverage between the carbides/nitrides and Co underlies the distinct reaction mechanisms observed for these catalysts under plasma conditions. These differences arise from distinct NH_2_* and NH* dehydrogenation barriers. As shown in Figure S21, Co_3_C­(001) exhibits a very high barrier for NH_2_* dehydrogenation and Co_3_N­(001) shows a very high barrier for NH* dehydrogenation. These high barriers, combined with the fast •NH_2_–N* formation pathway under plasma conditions prevent N* accumulation, resulting in consistently low N* surface coverages. In contrast, Co(0001) exhibits moderate activation barriers for both NH_2_* and NH* dehydrogenation. The fast kinetics of NH_2_* and NH* dehydrogenation combined with the slow N≡N coupling kinetics leads to the high N* coverage on Co(0001).

Surface charge analysis further explains these trends. Increasing positive surface charge systematically lowers NH_2_* dehydrogenation barriers, i.e., from 1.66 eV on Co_3_C to 1.12 eV on Co and further to 0.95 eV on Co_3_N (Figure S21a). Conversely, increasing positive charge raises NH* dehydrogenation barriers, i.e., from 1.10 eV on Co_3_C to 1.26 eV on Co and further to 1.43 eV on Co_3_N (Figure S21b). Thus, negatively charged Co_3_C suppresses NH_2_* dehydrogenation, while positively charged Co_3_N suppresses NH* dehydrogenation, yielding consistently low N* coverages that favor the radical-driven •NH_2_–N* pathway.

### 3.5 Plasma-Driven Reduction of Temperature Requirements for NH_3_ Decomposition

To further quantify how plasma activation lowers the temperature requirement for a certain catalytic activity, we compared the operating temperatures needed to achieve a target TOF of 5 s^–1^ across the catalysts examined (Figure 8). Under conventional thermal conditions, this reactivity requires high temperatures of 502 °C, 712 °C, 687 °C, and 864 °C for Co_3_C­(001), Co_3_N­(001), Ru(0001), and Co(0001), respectively. These elevated requirements reflect the fundamental kinetic limitations imposed by N≡N bond formation. Incorporating vibrational excitation under plasma conditions yields modest improvements, reducing the required temperatures to 460 °C, 674 °C, 681 °C, and 551 °C, respectively. These incremental reductions indicate that while vibrational excitation contributes additional activation channels, it has limited influence on the dominant reaction mechanism. Furthermore, the inclusion of plasma-generated reactive radical produce dramatic enhancements. The operating temperatures drop to 267 °C for Co_3_C­(001), 415 °C for Co_3_N­(001), 510 °C for Co(0001), while remaining the same at 681 °C for Ru(0001). This pronounced reduction for Co_3_C­(001) and Co_3_N­(001) is attributed to a fundamental shift in the reaction mechanism, where NH_3_^(v_1_)^ dissociation becomes the dominant rate-limiting step rather than N≡N bond formation. Consequently, employing plasma-active, cost-effective catalysts such as Co_3_C­(001) and Co_3_N­(001) instead of Ru(0001) typically used under thermal conditions, the operating temperature for NH_3_ decomposition can be reduced by up to ∼400 °C while maintaining desired TOFs, demonstrating the potential of plasma-catalyst coupling over carbides for energy-efficient hydrogen production.

**Figure 8:**
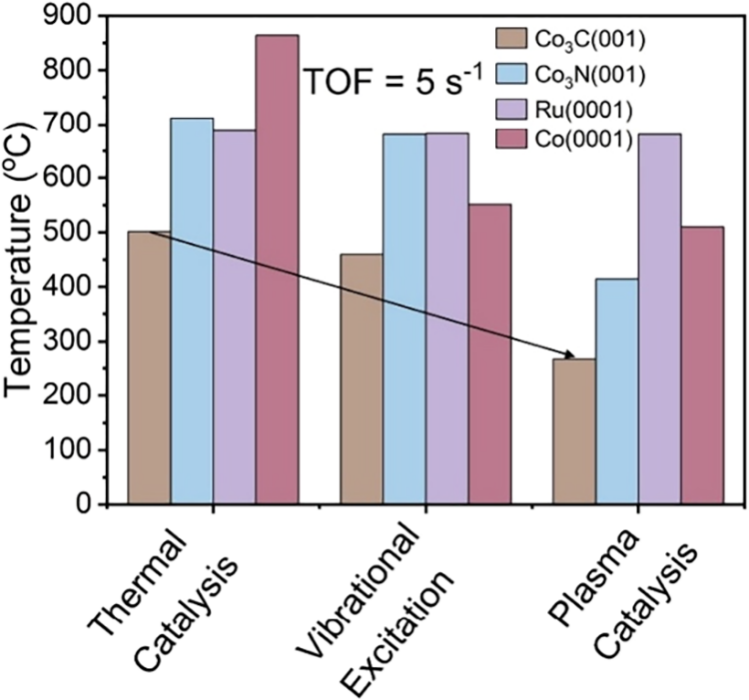
Required temperatures to achieve a target TOF of 5 s^–1^, across all catalysts examined under thermal conditions, plasma-induced vibrational excitation only conditions, and plasma-induced vibrational excitation and reactive radical interaction conditions.

## 4 Conclusions

This work establishes a comprehensive multiscale simulation framework that bridges zero-dimension plasma kinetics, DFT calculations, and mean-field microkinetic model to elucidate the mechanisms governing plasma-enhanced ammonia decomposition over transition metal carbides and nitrides. Under thermal conditions, Co_3_C­(001) is intrinsically the most active catalyst due to its negatively charged surface, strong N* binding, and correspondingly low N≡N formation barrier. Plasma activation introduces vibrationally excited NH_3_ and reactive radicals that bypass this thermal bottleneck: while vibrational excitation alone modestly enhances activity, the addition of radical-surface E-R interactions produces three to 6 orders of magnitude TOF enhancement on Co_3_C­(001) and Co_3_N­(001). This arises from a mechanistic shift in which a radical-driven •NH_2_–N* pathway becomes dominant for N_2_* formation and the RLS transitions from N≡N formation to NH_3_^(v_1_)^ dissociation, enabled by suppressed N* coverages on carbides and nitrides. As a result, the temperature required to reach a TOF of 5 s^–1^ drops by up to ∼400 °C relative to Ru-based thermal catalysis. Overall, this study provides molecular-level insights into plasma-catalyst coupling and offers guiding principles for designing energy-efficient, low-temperature, and cost-effective catalytic systems for hydrogen production from low-carbon ammonia.

## Supplementary Material



## Notes

Detailed descriptions of the DFT and MKM methodologies, adsorption and reaction energies, activation barriers, MKM parameters, plasma-activated species concentrations, and degree of rate control analyses (PDF)
