# Supplementary material for: Multi-Scale Modeling for Plasma-Enhanced Ammonia Decomposition over Carbides and Nitrides
Source: ACS Catal. 2025 Dec 19;16(1):717–28. doi: 10.1021/acscatal.5c07452 (PMC12772124; doi:10.1021/acscatal.5c07452)
Supplement: Supplementary file 1 [file cs5c07452_si_001.pdf]

Multi-Scale Modeling for Plasma-Enhanced Ammonia Decomposition over Carbides and Nitrides

Saleh Ahmat Ibrahim[1], Qiang Li[2], Fanglin Che[1]*
[1]Department of Chemical Engineering, Worcester Polytechnic Institute, Worcester, 01609, USA.
[2]Department of Chemical Engineering, University of Massachusetts Lowell, Lowell, 01854, USA.
*Corresponding authors: fche@wpi.edu

Table of Contents

1. Bulk Properties of Co_3C and Co_3N 2
2. Bulk Stability via First-Principles Phase Diagrams 4
3. Adsorption of Reaction Intermediates 6
4. Zero-Dimensional Plasma Kinetic Solver (ZDPlasKin) 16
5. Microkinetic Modeling 17
   5.1. Turnover Frequency (TOF) Calculation 18
   5.2. Degree of Rate Control (DRC) 19
   5.3. Influence of the Reverse Reaction in Plasma-Assisted NH_3 Decomposition 19
   5.4. Role of C and N under Plasma Conditions 22
References 23

1. Bulk Properties of Co_3C and Co_3N

Co_3C adopts a cementite-derived structure and crystallizes in the orthorhombic *Pnma* space group.[1] In this structure, carbon atoms occupy the 4c sites, while cobalt atoms are positioned at both 4c and 8d sites, as shown in **Figure S1a**.[2] In contrast, Co_3N exhibits an upper bainite structure, crystallizing in the hexagonal P6_322 space group, where nitrogen atoms occupy 2c sites, and cobalt atoms are located at 6g sites (**Figure S1b**).[3, 4] To investigate their surface properties, the stable bulk crystal structures of Co_3C and Co_3N were first optimized. The calculated lattice parameters of pure Co_3C and Co_3N are summarized in **Table S1,** which are in good agreement with other theoretical reported litearture.[1, 5]

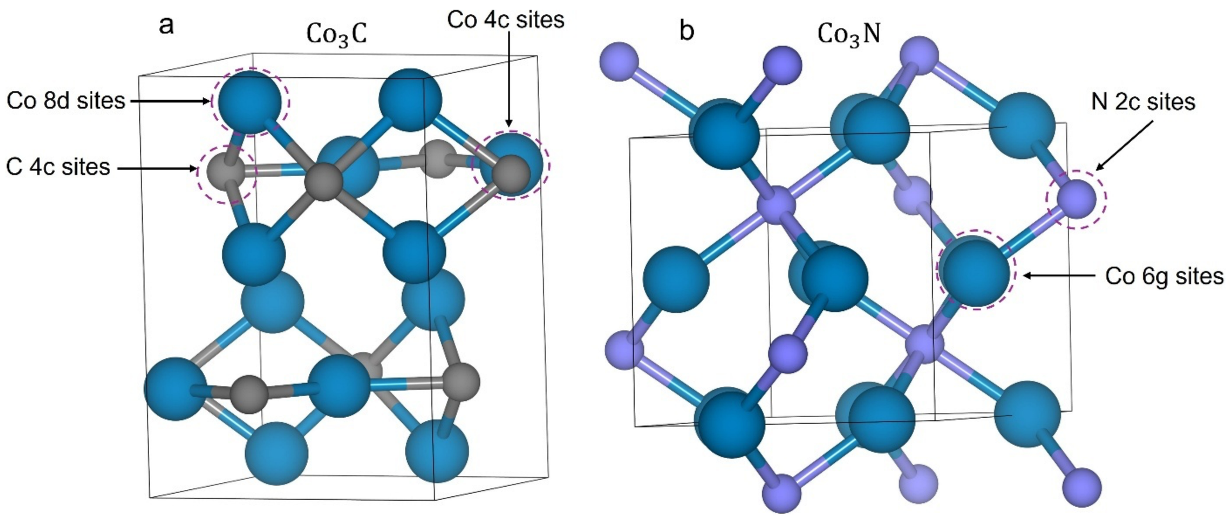

Figure S1. Crystal structures of (a) bulk orthorhombic Co_3C and (b) hexagonal Co_3N. Large blue spheres represent cobalt (Co) atoms; small gray spheres represent carbon (C) atoms in Co_3C; small purple spheres represent nitrogen (N) atoms in Co_3N.

Table S1. The calculated lattice parameters of Co_3C and Co_3N bulks.

		**Lattice Constants (Å)**					
Material	Crystal System	This work			Literature[1, 5]		
		**a**	**b**	**c**	**a**	**b**	**c**
Co_3C	Orthorhombic	4.393	4.919	6.594	4.483	5.033	6.731
Co_3N	Hexagonal	4.540	4.540	4.230	4.511	4.511	4.240

To identify the most stable phases for our study, we first collected all experimentally and/or computationally reported cobalt carbide and nitride structures available in the Materials Project database.[6] This includes Co_2C and Co_3C for carbides, and Co_2N, Co_3N, and Co_4N for nitrides. All bulk structures were fully optimized, and their formation enthalpies (ΔH) were evaluated using the following expressions:

For Co_3C (12 Co atoms and 4 C atoms):

$$\Delta H_{Co_3C} = \frac{E_{Co_3C} - 12 \cdot E_{Co} - 4 \cdot E_C}{16} \tag{S1}$$

For Co_3N (6 Co atoms and 2 N atoms):

$$\Delta H_{Co_3N} = \frac{E_{Co_3N} - 6 \cdot E_{Co} - 2 \cdot E_N}{8} \tag{S2}$$

where E_{Co3C} and E_{Co3N} are the total energies of the Co_3C and Co_3N bulk, respectively. E_{Co} is the total energy of a single Co atom in its pure bulk form, E_C is the total energy of a single C atom in pure graphite bulk and E_N is half of the total energy of nitrogen gas (N_2). A more negative formation enthalpy indicates a more thermodynamically stable bulk structure.

The corresponding formation enthalpies for all phases are summarized in **Figure S2**. As shown, that Co_3C has a more negative formation enthalpy than Co_2C, indicating that Co_3C is the more stable carbide phase. This agrees well with the reported formation enthalpy by Marshall et al..[7] It also identified Co_3N as the most stable nitride phase among Co_2N, Co_3N, and Co_4N. These fundamental thermodynamic trends guided our selection of Co_3C and Co_3N as the representative carbide and nitride phases for our study.

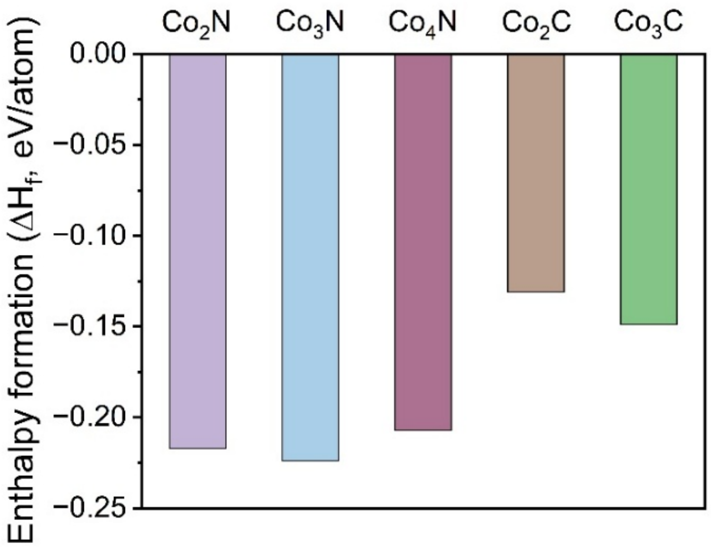

Figure S2. DFT-calculated formation enthalpies of the cobalt carbide (Co_2C and Co_3C) and nitride (Co_2N, Co_3N, and Co_4N) bulk phases.

Recent studies[8-10] on the cobalt carbide system show that Co_3C (cementite-type, Pnma) and Co_2C (Pnnm) are known as the two competitive low-energy cobalt carbides phases, with Co_3C exhibiting the more favorable formation enthalpy under thermodynamic conditions relevant to catalysis, specifically within a temperature range of 300-800 K and pressures spanning 0-50 GPa. High-pressure experiments[7] combined with first-principles calculations demonstrate that although Co_3C and Co_2C are close in formation energy at ambient conditions, increasing pressure makes the formation enthalpy of Co_3C progressively more favorable while Co_2C becomes less stable. This stabilization enables bulk synthesis of Co_3C and identifies it as a viable rare-earth-free magnetic material. Turgut et al.[11] used reactive ball-milling studies to show that Co_3C forms preferentially and possesses a higher decomposition temperature than Co_2C, indicating greater kinetic and thermal stability. Wet-chemistry synthesis routes[12] further confirm that although Co_3C is more challenging to synthesize, once formed it persists as a single, well-defined phase, whereas Co_2C often appears only within mixed-phase products unless tightly controlled.

For cobalt nitrides (Co_3N, Co_2N, Co_4N), to the best of our knowledge, no work explicitly benchmarks their thermodynamic stability against one another. However, several studies across electrocatalysis,[13] energy conversion,[14] and ammonia synthesis[15, 16] consistently employ Co_3N as the active phase rather than Co_2N or Co_4N. For example, Co_3N is investigated as a functional surface[13] in water splitting and oxygen evolution reaction, used as a high-performance electrode[14] for hydrazine oxidation and self-powered hydrogen production, and identified as a critical phase in ammonia synthesis mechanisms. The repeated selection of Co_3N in these applications highlights its practical synthesizability and stability compared to Co_2N and Co_4N, aligning with our theoretical finding that Co_3N is the most thermodynamic stable nitride phase.

2. Bulk Stability via First-Principles Phase Diagrams

To assess the stability of Co_3C and Co_3N under thermal and plasma conditions, we evaluated their bulk thermodynamics across hydrogen pressures generated during NH_3 decomposition using first-principles phase diagram calculations:

$$Co_3C(s) + 2H_2(g) \rightleftharpoons 3Co(s) + CH_4(g) \quad \text{(S3)}$$

$$Co_3N(s) + 1.5H_2(g) \rightleftharpoons 3Co(s) + NH_3(g) \quad \text{(S4)}$$

The corresponding decomposition energies are:

$$E_{Co\_3C} = 3E_{Co}^{bulk} + E_{CH_4} - E_{Co_3C}^{bulk} - 2E_{H_2} \quad \text{(S5)}$$

$$E_{Co\_3N} = 3E_{Co}^{bulk} + E_{NH_3} - E_{Co_3N}^{bulk} - \frac{3}{2}E_{H_2} \quad \text{(S6)}$$

where $E_{Co_3C}^{bulk}$, $E_{Co_3N}^{bulk}$ and E_{Co}^{bulk} are the electronic energies of Co_3C, Co_3N, and Co bulk systems, and E_{CH_4}, E_{NH_3} and E_{H_2} are the electronic energies of the corresponding gas molecules.

To include temperature and pressure effects, we then calculated Gibbs free energies of decomposition of Co_3C and Co_3N bulk systems as function of hydrogen chemical potentials:

$$\Delta G_{Co\_3C} = 3E_{Co}^{bulk} + \Delta\mu_{CH_4}(T, p) - E_{Co_3C}^{bulk} - 4\Delta\mu_H(T, p) \quad \text{(S7)}$$

$$\Delta G_{Co\_3N} = 3E_{Co}^{bulk} + \Delta\mu_{NH_3}(T, p) - E_{Co_3N}^{bulk} - 3\Delta\mu_H(T, p) \quad \text{(S8)}$$

The temperature- and pressure-dependent chemical potentials[17, 18] for methane, ammonia, and hydrogen were computed using:

$$\Delta\mu_{CH_4}(T,\, p) = E_{CH_4} + \Delta\mu_{CH_4}(T,\, p^o) + k_BTln\left(\frac{p_{CH_4}}{p^o}\right) \quad \text{(S9)}$$

$$\Delta\mu_{NH_3}(T,\, p) = E_{NH_3} + \Delta\mu_{NH_3}(T,\, p^o) + k_BTln\left(\frac{p_{NH_3}}{p^o}\right) \quad \text{(S10)}$$

$$\Delta\mu_{H}(T,\, p) = \frac{1}{2}E_{H_2} + \frac{1}{2}\Delta\mu_{H_2}(T,\, p^o) + \frac{1}{2}k_BTln\left(\frac{p_{H_2}}{p^o}\right) \quad \text{(S11)}$$

with $\Delta\mu_i(T,\, p^o)$ obtained from the NIST-JANAF thermochemical tables[19] under the ideal gas assumption.

Substituting these expressions yields the full temperature- and pressure-dependent Gibbs decomposition energies:

$$\Delta G_{Co\_3C} = E_{Co\_3C} + \Delta\mu_{CH_4}(T,\, p^o) - 2\Delta\mu_H(T,\, p^o) + k_BTln\left(\frac{p_{CH_4}}{p^o}\right) - 2k_BTln\left(\frac{p_{H_2}}{p^o}\right) \quad \text{(S12)}$$

$$\Delta G_{Co\_3N} = E_{Co\_3N} + \Delta\mu_{NH_3}(T,\, p^o) - \frac{3}{2}\Delta\mu_H(T,\, p^o) + k_BTln\left(\frac{p_{NH_3}}{p^o}\right) - \frac{3}{2}k_BTln\left(\frac{p_{H_2}}{p^o}\right) \quad \text{(S13)}$$

These expressions can access the bulk stability of Co_3C and Co_3N as a function of temperature, pressure, and hydrogen chemical potential (partial pressure).

Using the stoichiometry of ammonia decomposition ($2NH_3 \rightleftharpoons N_2 + 3H_2$), the corresponding hydrogen partial pressure as a function of NH_3 conversion is given by $p_{H_2} = \frac{1.5X}{1+X}$. From our previous experimental measurements[20] at 400 °C, Ru and Co achieved thermal conversions of 7.9% and 1.8%, respectively, while literature reports[21-23] have slightly higher thermal conversions of approximately 10-20% at this temperature. These conversion levels translate to hydrogen partial pressures below 0.25 bar, with early-stage thermal decomposition corresponding to pressures as low as 10^{-4} bar; therefore, we selected 10^{-4}-0.25 bar as the representative thermal H_2-pressure range.

Under plasma-assisted conditions at 400 °C, our experiments[20] show that Ru reaches 58.3% conversion and Co reaches 22.8%, and literature reports[24-26] conversions ranging from 20-65% depending on plasma power and reactor configuration. These values correspond to hydrogen partial pressures of roughly 0.25-0.60 bar, which we adopt as the representative range for plasma-enhanced ammonia decomposition. As a result, a combined hydrogen-pressure window of 10^{-4} to 0.60 bar is used to capture both thermal conditions, where H_2 pressures remain small (≈10^{-4}-0.25 bar), and plasma-assisted conditions, where vibrationally and radical-activated pathways substantially enhance NH_3 dissociation and increase H_2 formation (0.25-0.60 bar).

Using these pressure ranges, we computed the Gibbs free energies (ΔG) for Co_3C and Co_3N bulk decomposition as a function of $\Delta\mu_H$, covering H_2 partial pressures from 10^{-4} to 0.6 bar, representative of both thermal and plasma-assisted NH_3 decomposition at 400 °C. As shown in **Figure S3a**, both Co_3C and Co_3N exhibit positive Gibbs free energies ($\Delta G > 0$). Although ΔG decreases as $\Delta\mu_H$ increases, it remains positive for all relevant thermal and plasma conditions, indicating that both Co_3C and Co_3N are thermodynamically stable and unfavorable to be decomposed under thermal and plasma conditions during ammonia decomposition at 400 °C.

Figure S3b shows the Gibbs free energy of bulk decomposition as a function of temperature at fixed hydrogen chemical potential (0.75 bar). We use a hydrogen partial pressure of 0.75 bar because it represents the maximum value achievable under fully decomposed ammonia for both thermal and plasma conditions at ambient pressure. For both Co_3C and Co_3N, ΔG remains positive across 400-800 °C, decreasing from ~0.62 eV at 400 °C to ~0.10 eV at 800

°C. Although elevated temperatures reduce the thermodynamic stability, the free energy remains positive, indicating that neither phase becomes thermodynamically prone to bulk decomposition within the temperature range relevant to NH_3 decomposition. This further confirms that Co_3C and Co_3N remain stable under both thermal and plasma conditions.

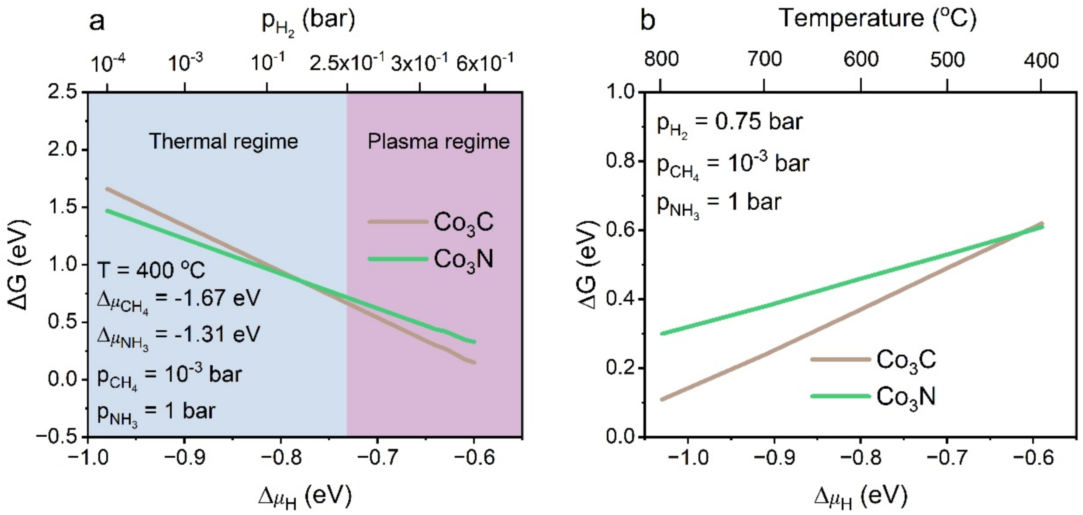

Figure S3. (a) Gibbs free energies (ΔG) of Co_3C and Co_3N bulk systems as a function of hydrogen chemical potential (partial pressure) at 400 °C, obtained via first-principles phase diagram. (b) Gibbs free energy of bulk Co_3C and Co_3N at 0.75 bar of hydrogen partial pressure as a function of temperature.

3. Adsorption of Reaction Intermediates

The most stable facets, C-terminated Co_3C(001), N-terminated Co_3N(001), Ru(0001), and Co(0001) were selected to perform DFT calculations (**Figure S4**). On the Co_3C(001) surface, six adsorption sites were identified: the top of a first-layer Co atom (top-1), the top of a subsurface Co atom (top-2), the top of a surface C atom (top-3), short bridge between Co-Co atoms (bri-1), long bridge between Co-Co atoms (bri-2), and a threefold Co_3-hollow site with a subsurface Co atom (hollow). The Co_3N(001) surface exhibited seven adsorption sites: top of Co (top-1), top of N (top-2), short bridge between Co-Co atoms (bri-1), long bridge between Co-Co atoms (bri-2), a Co_3-hollow site with a subsurface Co atom (hcp), a Co_3-hollow site with a subsurface N atom in the second layer (fcc-1), and a Co_3-hollow site without a subsurface atom (fcc-2). On the Ru(0001) and Co(0001) surfaces, four adsorption sites were identified: top of Ru or Co (top), bridge between Ru-Ru or Co-Co atoms (bri), Ru_3- or Co_3-hollow site with a subsurface Ru or Co atom (hcp), and Ru_3- or Co_3-hollow site without a subsurface atom (fcc).

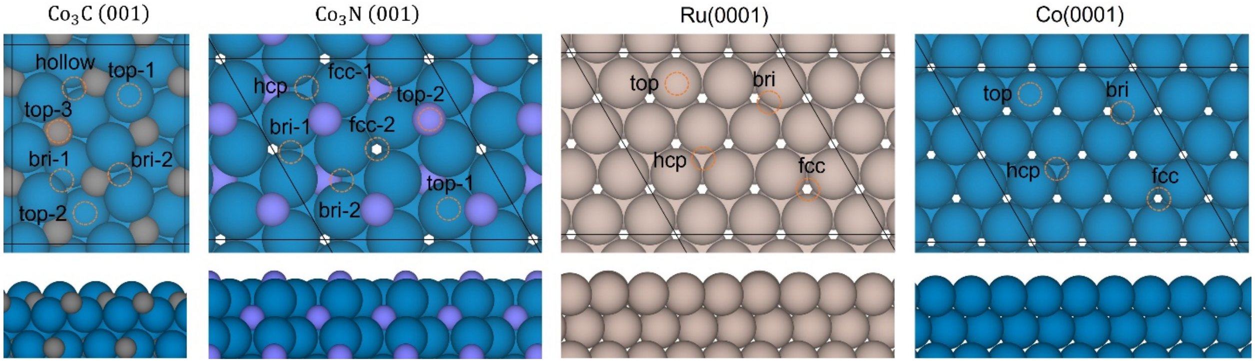

Figure S4. Top and side views of $Co_3C(001)$, $Co_3N(001)$, Ru(0001), and Co(0001) surfaces. Possible adsorption sites are marked with orange dashed circles. The $Co_3C(001)$ surface features six adsorption sites, $Co_3N(001)$ has seven adsorption sites, and Ru(0001) and Co(0001) have four adsorption sites. Identified sites include top (Co, C, N), bridge, and hollow sites. Grayish orange spheres represent Ruthenium (Ru) atoms.

Adsorption energy[27, 28] (E_{ad}) for reactive intermediates such as NH_3^*, NH_2^*, NH^*, N^*, H^*, NH_2-NH_2^*, NH_2-NH^*, NH_2-N^*, NH-NH^* and NH-N^* are calculated as follows:

$$E_{ad}^{i} = \mathrm{E}_{ads+\mathrm{Slab}} - \mathrm{E}_{\mathrm{Slab}} - \mathrm{E}_{(ads)_{\mathrm{gas}}} \tag{S14}$$

where $E_{ads+Slab}$ is the total electronic energy of the adsorbate-slab system, E_{Slab} is the energy of the clean slab, and $E_{(ads)gas}$ represents gas-phase energy of the adsorbate.

Adsorption energy calculations were performed to identify the most favorable configurations of NH_3 decomposition related intermediates. The optimized geometries and corresponding adsorption energies for each configuration are shown in **Figures S5-S14**.

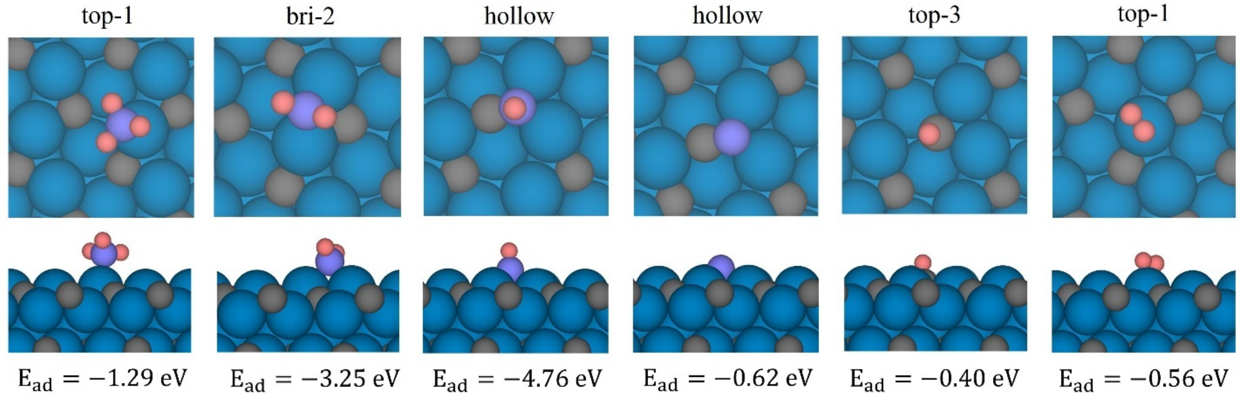

Figure S5. Top and side views of NH_3^*, NH_2^*, NH^*, N^*, H^*, and H_2^* adsorbed at their most stable configurations on $Co_3C(001)$, along with their corresponding adsorption energies. NH_3^* and H_2^* preferentially adsorb at the top-1 site, NH_2^* binds most favorably at the bri-2 site, NH^* and N^* favor the hollow sites. H^* adsorption is most stable on the top-3 site. In contrast, NH_3^* and H_2^* adsorb unfavorably on the top-2, top-3, bri-1, bri-2, and hollow sites. NH_2^* binds unfavorably on the top-1, top-2, top-3, bri-1, and hollow sites. Similarly, NH^* and N^* are unstable on the top-1, top-2, top-3, bri-1, and bri-2 sites, whereas H^* adsorption is unfavorable on the top-1, top-2, bri-1, bri-2, and hollow sites. On these unfavorable sites, the adsorbates either exhibit very weak binding or migrate to nearby stable configurations. Large blue spheres represent cobalt (Co) atoms; small gray spheres represent carbon (C) atoms in Co_3C; small purple spheres represent nitrogen (N); small pink spheres represent hydrogen (H) atoms.

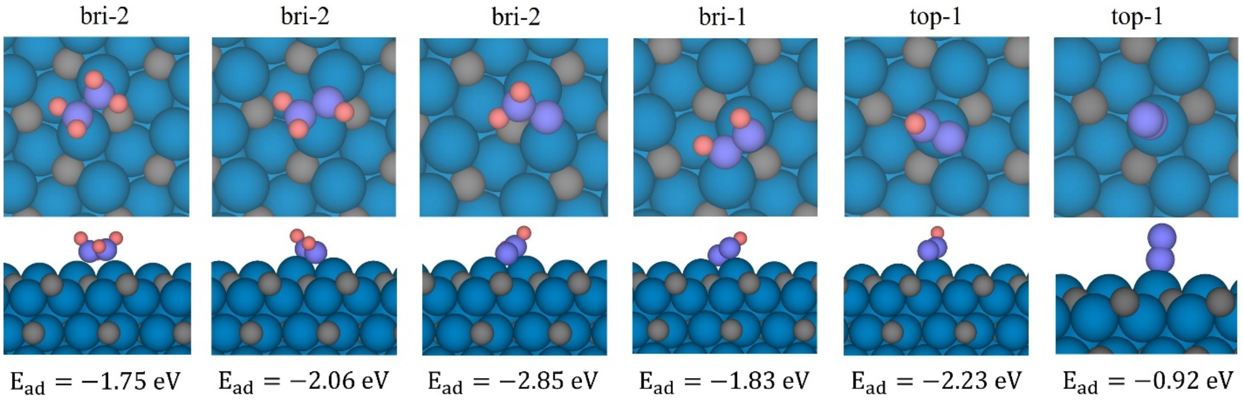

Figure S6. Top and side views of NH_2-NH_2^*, NH_2-NH^*, NH_2-N^*, NH-NH^*, NH-N^*, and N_2^* adsorbed at their most stable configurations on $Co_3C(001)$, along with their corresponding adsorption energies. NH_2-NH_2^*, NH_2-NH^* and NH_2-N^* preferentially bind at the bri-2 sites, NH-NH^* favors the bri-1 site, while NH-N^*, and N_2^* are most stable on top-1 sites. In contrast, NH_2-NH_2^*, NH_2-NH^* and NH_2-N^* adsorb unfavorably on the top-1, top-2, top-3, bri-1, and hollow sites. NH-NH^* exhibits unfavorable adsorption on the top-1, top-2, top-3, bri-2, and hollow sites, whereas NH-N^* and N_2^* bind unfavorably on the top-2, top-3, bri-1, bri-2, and hollow sites. On these unfavorable sites, each adsorbate either adsorbs weakly or migrates to the nearest stable site.

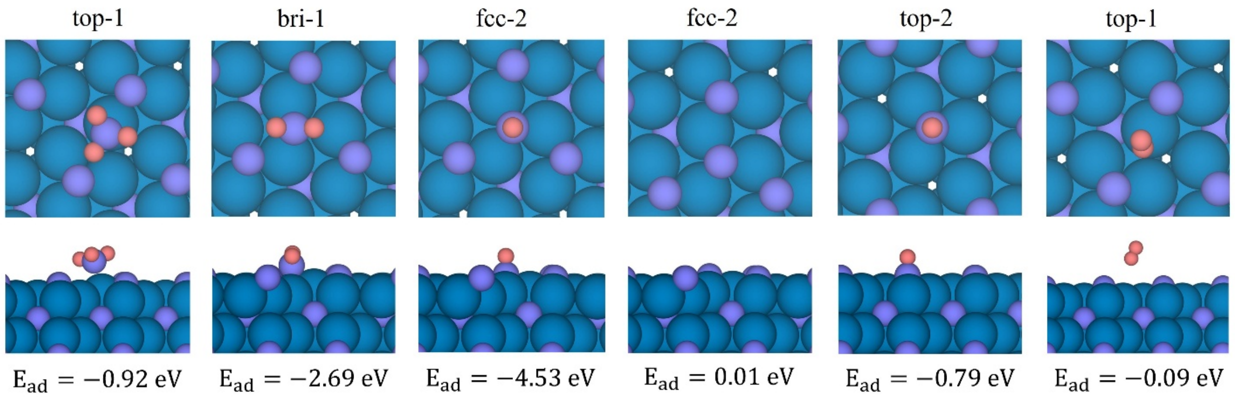

Figure S7. Top and side views of NH_3^*, NH_2^*, NH^*, N^*, H^*, and H_2^* adsorbed at their most stable configurations on $Co_3N(001)$, along with their corresponding adsorption energies. NH_3^* and H_2^* prefer adsorption on the top-1, NH_2^* binds most favorably at the bri-1, NH^* and N^* favor fcc-2 sites, while H^* binds preferentially on the top-2 site. In contrast, NH_3^* and H_2^* adsorb unfavorably on the top-2, bri-1, bri-2, hcp, fcc-1, and fcc-2 sites. NH_2^* binds unfavorably on the top-1, top-2, bri-2, hcp, fcc-1, and fcc-2 sites. NH^* and N^* species exhibit unfavorable adsorption on the top-1, top-2, bri-1, bri-2, hcp, and fcc-1 sites, whereas H^* binds unfavorably on the top-1, bri-1, bri-2, hcp, fcc-1, and fcc-2 sites. On these unfavorable sites, each adsorbate either adsorbs weakly or migrates to the nearest stable site.

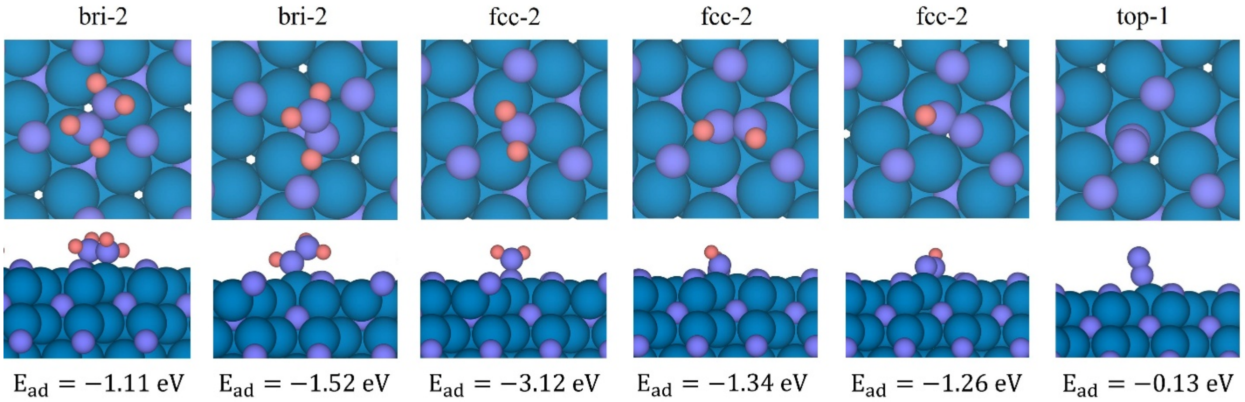

Figure S8. Top and side views of NH_2-NH_2^*, NH_2-NH^*, NH_2-N^*, NH-NH^*, NH-N^*, and N_2^* adsorbed at their most stable configurations on $Co_3N(001)$, along with their corresponding adsorption energies. NH_2-NH_2^* and NH_2-NH^* preferentially adsorb at the bri-2 sites, NH_2-N^*, NH-NH^*, and NH-N^* favor fcc-2 sites, while N_2^* are most stable on top-1 sites. In contrast, NH_2-NH_2^* and NH_2-NH^* adsorb unfavorably on the top-1, top-2, bri-1, hcp, fcc-1, and fcc-2 sites. NH_2-N^*, NH-NH^* and NH-N^* species exhibit unfavorable adsorption at the top-1, top-2, bri-1, bri-2, hcp, and fcc-1 sites, whereas N_2^* binds unfavorably on the top-2, bri-1, bri-2, hcp, fcc-1, and fcc-2 sites. On these unfavorable sites, each adsorbate either adsorbs weakly or migrates to the nearest stable site.

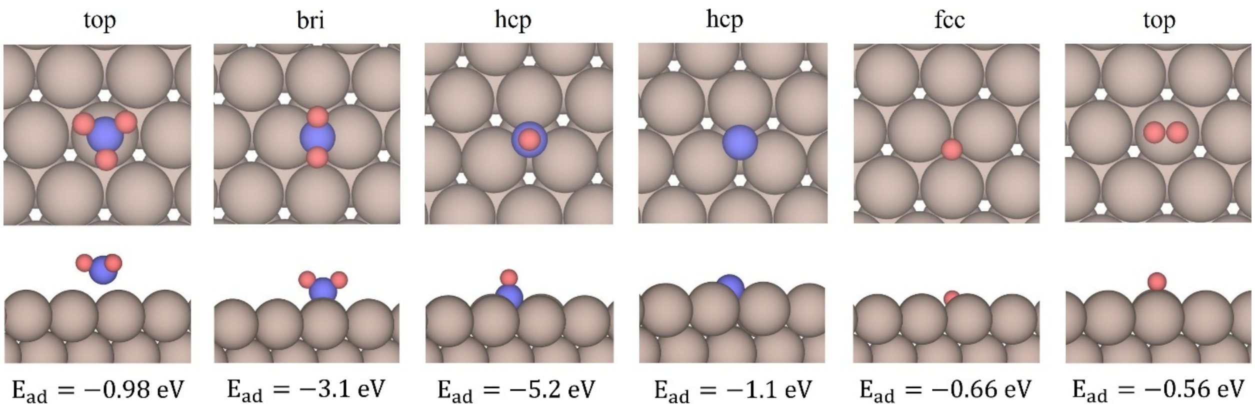

Figure S9. Top and side views of NH_3^*, NH_2^*, NH^*, N^*, H^*, and H_2^* adsorbed at their most stable configurations on Ru(0001), along with their corresponding adsorption energies. NH_3^* and H_2^* prefer adsorption on the top of Ru, NH_2^* binds most favorably at the bridge, NH^* and N^* favor hcp sites, while H^* adsorbs preferentially at the fcc site. In contrast, NH_3^* and H_2^* adsorb unfavorably on the bri, fcc, and hcp sites. NH_2^* binds unfavorably at the top, fcc, and hcp sites. NH^* and N^* species exhibit unfavorable adsorption at the top, bri, and fcc sites, whereas H^* binds unfavorably on the top, bri, and hcp sites. On these unfavorable sites, each adsorbate either adsorbs weakly or migrates to the nearest stable site.

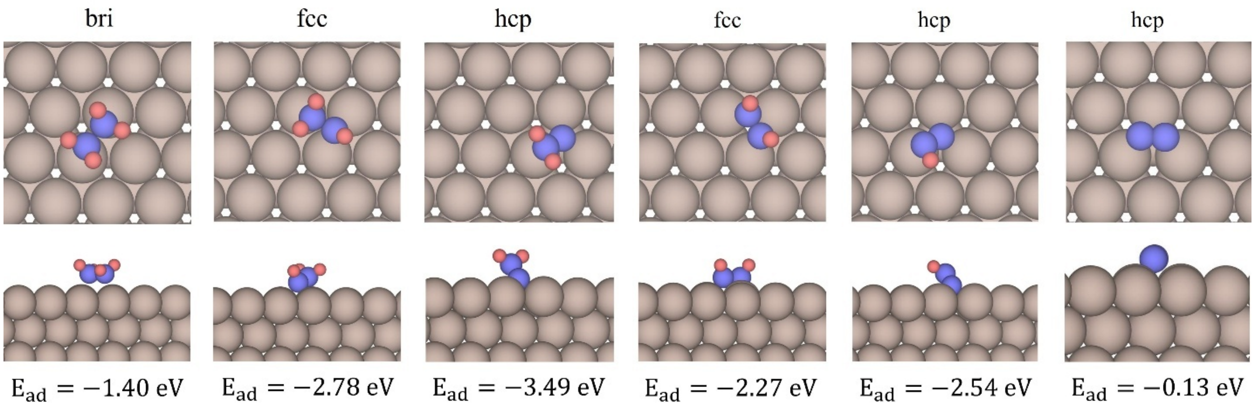

Figure S10. Top and side views of NH_2-NH_2^*, NH_2-NH^*, NH_2-N^*, NH-NH^*, NH-N^*, and N_2^* adsorbed at their most stable configurations on Ru(0001), along with their corresponding adsorption energies. NH_2-NH_2^* preferentially adsorbs at the bri site, NH_2-NH^* and NH-NH^* favor fcc sites, while NH_2-N^*, NH-N^*, and N_2^* are most stable the hcp sites. In contrast, NH_2-NH_2^* adsorbs unfavorably on the top, fcc, and hcp sites. NH_2-NH^* and NH-NH^* species exhibit unfavorable adsorption on the top, bri, and hcp sites, whereas NH_2-N^*, NH-N^*, and N_2^* bind unfavorably on the top, bri, and fcc sites. On these unfavorable sites, each adsorbate either adsorbs weakly or migrates to the nearest stable site.

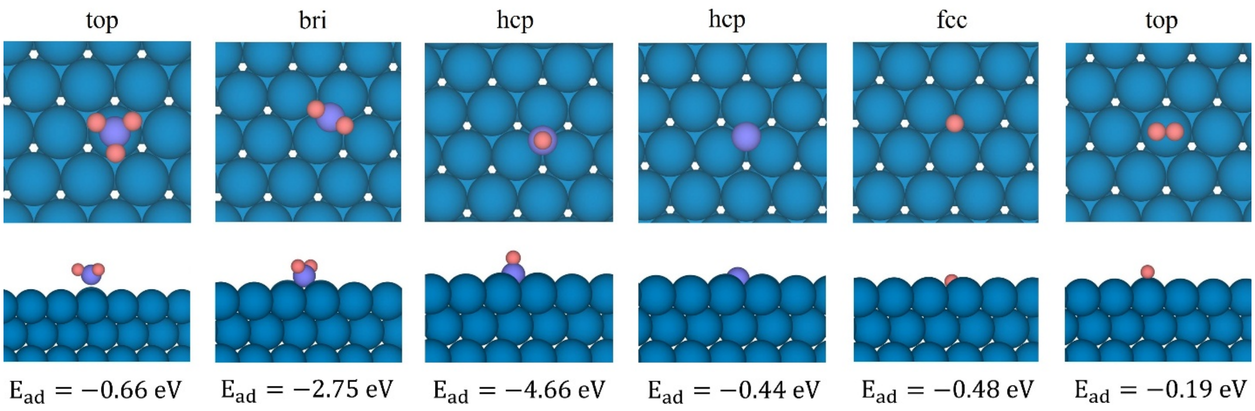

Figure S11. Top and side views of NH_3^*, NH_2^*, NH^*, N^*, H^*, and H_2^* adsorbed at their most stable configurations on Co(0001), along with their corresponding adsorption energies. NH_3^* and H_2^* prefer adsorption on the top of Co, NH_2^* binds most favorably at the bridge, NH^* and N^* favor hcp sites, while H^* adsorbs preferentially at the fcc site. In contrast, NH_3^* and H_2^* adsorb unfavorably on the bri, fcc, and hcp sites. NH_2^* binds unfavorably at the top, fcc, and hcp sites. NH^* and N^* species exhibit unfavorable adsorption at the top, bri, and fcc sites, whereas H^* binds unfavorably on the top, bri, and hcp sites. On these unfavorable sites, each adsorbate either adsorbs weakly or migrates to the nearest stable site.

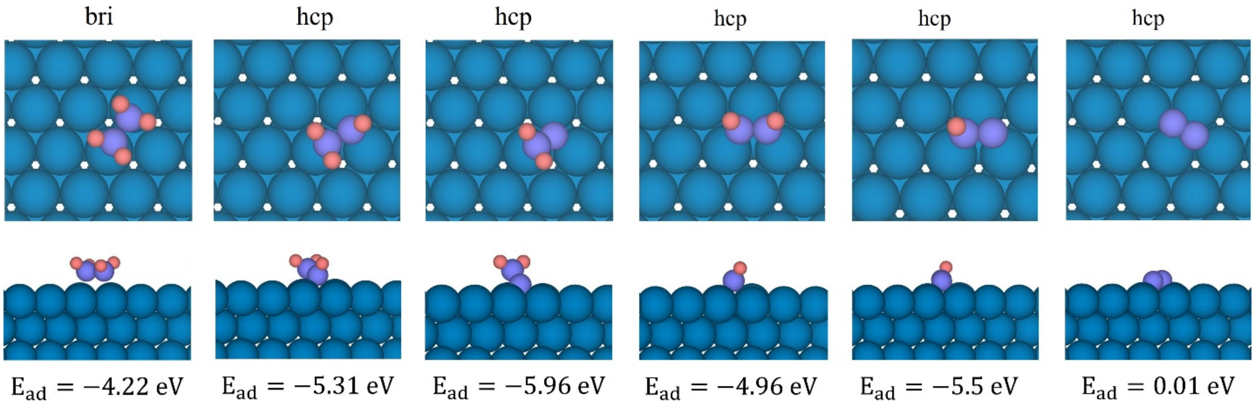

Figure S12. Top and side views of NH_2-NH_2^*, NH_2-NH^*, NH_2-N^*, NH-NH^*, NH-N^*, and N_2^* adsorbed at their most stable configurations on Co(0001), along with their corresponding adsorption energies. NH_2-NH_2^* preferentially adsorbs at the bri site, NH_2-NH^*, NH-NH^*, NH_2-N^*, NH-N^*, and N_2^*favor hcp sites. In contrast, NH_2-NH_2^* adsorbs unfavorably on the top, fcc, and hcp sites. NH_2-NH^*, NH-NH^*, NH_2-N^*, NH-N^*, and N_2^* species exhibit unfavorable adsorption on the top, bri, and fcc sites. On these unfavorable sites, each adsorbate either adsorbs weakly or migrates to the nearest stable site.

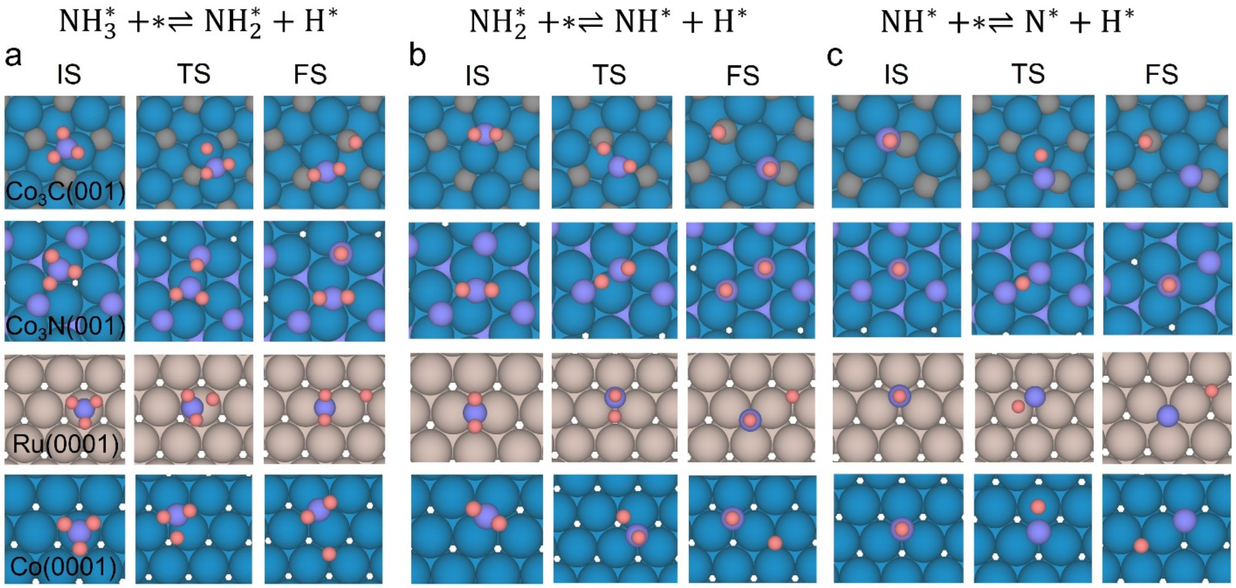

Figure S13. Initial state (IS), transition state (TS) and final state (FS) configurations for NH_x dehydrogenation steps over $Co_3C(001)$, $Co_3N(001)$, Ru(0001), and Co(0001) surfaces. The reactions include (a) $NH_3^* + * \rightleftharpoons NH_2^* + H^*$, (b) $NH_2^* + * \rightleftharpoons NH^* + H^*$, and (c) $NH^* + * \rightleftharpoons N^* + H^*$.

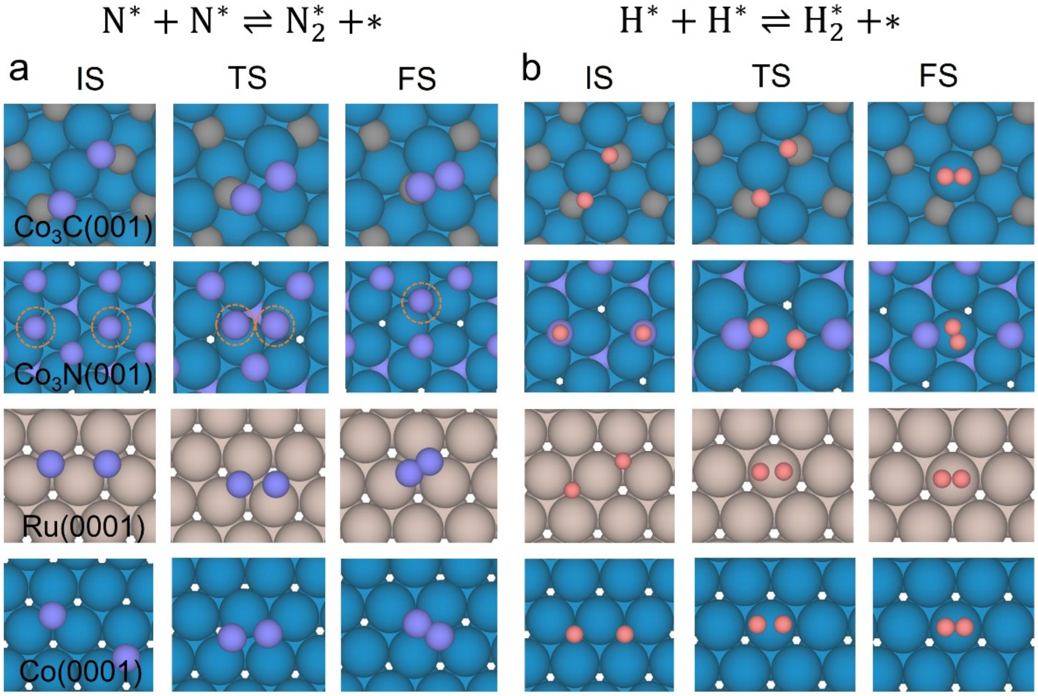

Figure S14. Initial state (IS), transition state (TS) and final state (FS) configurations for N≡N and H-H reactions over $Co_3C(001)$, $Co_3N(001)$, Ru(0001), and Co(0001) surfaces. The reactions include (a) $N^* + N^* \rightleftharpoons N_2^* + *$ and (b) $H^* + H^* \rightleftharpoons H_2^* + *$.

Table S2. Reaction energies and activation barriers (in eV) for the elementary steps considered in the microkinetic models. The dataset includes vibrationally excited NH_3 molecules, dissociative adsorption, molecular and radical adsorption, NH_x dehydrogenation via Langmuir-Hinshelwood (L-H) pathways, N≡N and H-H bond formation, and product desorption on $Co_3C(001)$, $Co_3N(001)$, Ru(0001), and Co(0001) surfaces.

Reaction	Reaction energy (eV)				Activation barrier (eV)			
	Co_3C	Co_3N	Ru	Co	Co_3C	Co_3N	Ru	Co
**Vibrational excitation**								
$NH_3^{(v_0)} + 2^* \rightleftharpoons NH_2^* + H^*$	-1.04	-0.86	-1.12	-0.60	1.1	1.0	1.29	1.14
$NH_3^{(v_1)} + 2^* \rightleftharpoons NH_2^* + H^*$	-1.68	-1.51	-1.76	-1.52	0.89	0.78	1.07	0.78
$NH_3^{(v_2)} + 2^* \rightleftharpoons NH_2^* + H^*$	-2.88	-2.70	-2.96	-2.67	0.48	0.36	0.65	0.32
$NH_3^{(v_3)} + 2^* \rightleftharpoons NH_2^* + H^*$	-4.16	-3.98	-4.24	-3.75	0.04	0.0	0.21	0.0
$NH_3^{(v_4)} + 2^* \rightleftharpoons NH_2^* + H^*$	-5.93	-5.76	-6.01	-4.78	0.0	0.0	0.0	0.0
**Adsorption**								
$NH_3(g) + * \rightleftharpoons NH_3^*$	-1.29	-0.91	-1.00	-0.66	0.0	0.0	0.0	0.0
**L-H NH_x dehydrogenation**								
$NH_3^* + * \rightleftharpoons NH_2^* + H^*$	0.26	0.05	-0.12	0.07	1.27	1.17	1.46	1.35
$NH_2^* + * \rightleftharpoons NH^* + H^*$	0.14	-0.59	-0.69	-0.34	1.66	0.95	1.03	1.12
$NH^* + * \rightleftharpoons N^* + H^*$	0.14	0.14	-0.18	0.21	1.10	1.43	1.07	1.26
**N≡N and H-H bond formation**								
$N^* + N^* \rightleftharpoons N_2^* + *$	0.33	0.11	1.90	0.75	1.82	2.45	2.18	1.97
$H^* + H^* \rightleftharpoons H_2^* + *$	0.23	1.50	0.67	0.76	0.91	1.50	0.84	0.86
**Desorption**								
$N_2^* \rightleftharpoons N_2(g) + *$	0.92	-0.13	0.10	-0.01	0.0	0.0	0.0	0.0
$H_2^* \rightleftharpoons H_2(g) + *$	0.56	0.09	0.55	0.19	0.0	0.0	0.0	0.0

Table S3. Reaction energies of elementary steps incorporated into microkinetic models, capturing radical-surface interactions through Eley-Rideal (E-R) mechanisms on $Co_3C(001)$, $Co_3N(001)$, Ru(0001), and Co(0001) surfaces. The reported steps are obtained under the key assumptions outlined in the manuscript.

Reaction No	Reaction	Reaction energy (eV)			
		Co_3C	Co_3N	Ru	Co
1	$\bullet NH_2 + * \rightleftharpoons NH_2*$	-3.25	-2.68	-3.12	-2.75
2	$\bullet NH + * \rightleftharpoons NH*$	-4.76	-4.53	-5.25	-4.66
3	$\bullet N + * \rightleftharpoons N*$	-5.82	-5.18	-6.40	-5.55
4	$\bullet H + * \rightleftharpoons H*$	-2.66	-3.06	-2.88	-2.74
5	$\bullet NH_2 + NH_3* \rightleftharpoons NH_2* + NH_3(g)$	-1.96	-1.77	-2.12	-2.08
6	$\bullet NH_2 + NH_2* \rightleftharpoons NH* + NH_3*$	-3.37	-3.33	-3.70	-3.15
7	$\bullet NH_2 + NH_2* \rightleftharpoons NH_2\text{-}NH_2*$	-1.72	-1.65	-1.51	-1.34
8	$\bullet NH_2 + NH_2* \rightleftharpoons NH* + NH_3(g)$	-2.08	-2.42	-2.70	-2.49
9	$\bullet NH_2 + NH* \rightleftharpoons NH_2\text{-}NH*$	-1.24	-0.93	-1.47	-1.69
10	$\bullet NH_2 + NH* \rightleftharpoons N* + NH_3(g)$	-2.08	-1.68	-2.18	-1.94
11	$\bullet NH_2 + N* \rightleftharpoons NH_2\text{-}N*$	-1.50	-2.40	-1.54	-1.69
12	$\bullet NH_2 + H* \rightleftharpoons NH_3*$	-3.51	-2.74	-3.00	-2.81
13	$\bullet NH_2 + H* \rightleftharpoons NH_3(g) + *$	-2.22	-1.83	-2.00	-2.15
14	$\bullet NH + NH_3* + * \rightleftharpoons NH_2* + NH_2*$	-4.65	-3.88	-4.67	-4.25
15	$\bullet NH + NH_2* \rightleftharpoons NH_2\text{-}NH*$	-2.75	-2.77	-3.60	-2.81
16	$\bullet NH + NH_2* \rightleftharpoons \bullet NH_2 + NH*$	-1.51	-1.84	-2.13	-1.91
17	$\bullet NH + NH* \rightleftharpoons NH\text{-}NH*$	-2.91	-2.65	-2.86	-2.94
18	$\bullet NH + NH* \rightleftharpoons \bullet NH_2 + N*$	-1.51	-1.11	-1.61	-1.36
19	$\bullet NH + N* \rightleftharpoons NH\text{-}N*$	-3.20	-2.86	-2.92	-3.17
20	$\bullet NH + H* \rightleftharpoons NH_2*$	-4.91	-3.94	-4.55	-4.31
21	$\bullet NH + H* \rightleftharpoons \bullet NH_2 + *$	-1.65	-1.25	-1.43	-1.57
22	$\bullet N + NH_3* + * \rightleftharpoons NH_2* + NH*$	-5.70	-5.27	-6.34	-5.69
23	$\bullet N + NH_2* \rightleftharpoons NH_2\text{-}N*$	-4.06	-4.90	-4.83	-4.49
24	$\bullet N + NH_2* \rightleftharpoons \bullet NH + NH*$	-1.05	-1.39	-1.67	-1.45
25	$\bullet N + NH* \rightleftharpoons NH\text{-}N*$	-4.25	-3.52	-4.07	-4.07
26	$\bullet N + NH* \rightleftharpoons \bullet NH + N*$	-1.05	-0.66	-1.15	-0.89
27	$\bullet N + N* \rightleftharpoons N_2*$	-5.49	-5.07	-4.09	-4.80
28	$\bullet N + H* \rightleftharpoons NH*$	-5.96	-5.33	-6.23	-1.10
29	$\bullet N + H* \rightleftharpoons \bullet NH + *$	-1.20	-0.80	-0.98	-5.80
30	$\bullet H + NH_3* \rightleftharpoons NH_2* + H_2(g)$	-1.61	-1.42	-1.77	-1.70
31	$\bullet H + NH_2* \rightleftharpoons NH* + H_2(g)$	-1.73	-2.07	-2.35	-2.10
32	$\bullet H + NH_2* \rightleftharpoons NH_3*$	-2.92	-3.12	-2.77	-2.80
33	$\bullet H + NH* \rightleftharpoons N* + H_2(g)$	-1.73	-1.33	-1.83	-1.60
34	$\bullet H + NH* \rightleftharpoons NH_2*$	-2.81	-2.47	-2.19	-2.40
35	$\bullet H + N* \rightleftharpoons NH*$	-2.81	-3.20	-2.71	-3.00
36	$\bullet H + H* \rightleftharpoons H_2*$	-2.43	-1.56	-2.21	-2.00
37	$\bullet H + H* \rightleftharpoons H_2(g)$	-1.87	-1.47	-1.65	-1.80

Table S4. Reaction energies of elementary steps incorporated into microkinetic models, including the refined radical-surface interactions via Eley-Rideal (E-R) mechanisms N_2H_x dehydrogenation pathways on Co_3C(001), Co_3N(001), Ru(0001), and Co(0001) surfaces.

No	Reaction	Reaction energy (eV)				Activation barrier (eV)			
		Co_3C	Co_3N	Ru	Co	Co_3C	Co_3N	Ru	Co
**Refined E-R mechanisms**									
1	$\bullet NH_2 + * \rightleftharpoons NH_2*$	-3.25	-2.68	-3.12	-2.75	0.0	0.0	0.0	0.0
2	$\bullet NH + * \rightleftharpoons NH*$	-4.76	-4.53	-5.25	-4.66	0.0	0.0	0.0	0.0
3	$\bullet N + * \rightleftharpoons N*$	-5.82	-5.18	-6.40	-5.55	0.0	0.0	0.0	0.0
4	$\bullet H + * \rightleftharpoons H*$	-2.66	-3.06	-2.88	-2.74	0.0	0.0	0.0	0.0
5	$\bullet NH_2 + NH_2* \rightleftharpoons NH* + NH_3*$	-3.37	-3.33	-3.70	-3.15	0.0	0.0	0.0	0.0
6	$\bullet NH_2 + NH_2* \rightleftharpoons NH_2\text{-}NH_2*$	-1.72	-1.65	-1.51	-1.34	0.0	0.0	0.0	0.0
7	$\bullet NH_2 + N* \rightleftharpoons NH_2\text{-}N*$	-1.50	-2.40	-1.54	-1.69	0.0	0.0	0.0	0.0
8	$\bullet NH + NH_3* + * \rightleftharpoons NH_2* + NH_2*$	-4.65	-3.88	-4.67	-4.25	0.0	0.0	0.0	0.0
9	$\bullet NH + NH_2* \rightleftharpoons NH_2\text{-}NH*$	-2.75	-2.77	-3.60	-2.81	0.0	0.0	0.0	0.0
10	$\bullet NH + N* \rightleftharpoons NH\text{-}N*$	-3.20	-2.86	-2.92	-3.17	0.0	0.0	0.0	0.0
11	$\bullet NH + H* \rightleftharpoons NH_2*$	-4.91	-3.94	-4.55	-4.31	0.0	0.0	0.0	0.0
12	$\bullet N + NH_3* + * \rightleftharpoons NH_2* + NH*$	-5.70	-5.27	-6.34	-5.69	0.0	0.0	0.0	0.0
13	$\bullet N + NH_2* \rightleftharpoons NH_2\text{-}N*$	-4.06	-4.90	-4.83	-4.49	0.0	0.0	0.0	0.0
14	$\bullet N + NH* \rightleftharpoons NH\text{-}N*$	-4.25	-3.52	-4.07	-4.07	0.0	0.0	0.0	0.0
15	$\bullet N + N* \rightleftharpoons N_2*$	-5.49	-5.07	-4.09	-4.80	0.0	0.0	0.0	0.0
16	$\bullet N + H* \rightleftharpoons NH*$	-5.96	-5.33	-6.23	-1.10	0.0	0.0	0.0	0.0
17	$\bullet H + NH* \rightleftharpoons NH_2*$	-2.81	-2.47	-2.19	-2.40	0.0	0.0	0.0	0.0
18	$\bullet H + N* \rightleftharpoons NH*$	-2.81	-3.20	-2.71	-3.00	0.0	0.0	0.0	0.0
19	$\bullet H + H* \rightleftharpoons H_2*$	-2.43	-1.56	-2.21	-2.00	0.0	0.0	0.0	0.0
**Langmuir-Hinshelwood (L-H) N_2H_x dehydrogenation**									
20	$NH_2\text{-}NH_2* \rightleftharpoons NH_2\text{-}NH* + H*$	0.62	0.13	-0.66	0.10	1.33	0.56	0.90	0.95
21	$NH_2\text{-}NH* \rightleftharpoons NH_2\text{-}N* + H*$	-0.11	-1.32	-0.25	-0.58	1.13	0.40	1.41	1.36
22	$NH_2\text{-}NH* \rightleftharpoons NH\text{-}NH* + H*$	-0.02	-0.46	0.04	-0.47	0.84	0.26	1.29	1.44
23	$NH\text{-}NH* \rightleftharpoons NH\text{-}N* + H*$	-0.15	-0.07	-0.23	-0.02	0.93	0.66	1.03	1.14
24	$NH_2\text{-}N* \rightleftharpoons NH\text{-}N* + H*$	-0.05	0.79	0.06	0.08	1.48	0.54	1.10	1.11
25	$NH\text{-}N* \rightleftharpoons N_2* + H*$	-1.09	-1.41	-0.19	-0.52	1.62	0.61	0.97	0.96

4. Zero-Dimensional Plasma Kinetic Solver (ZDPlasKin)

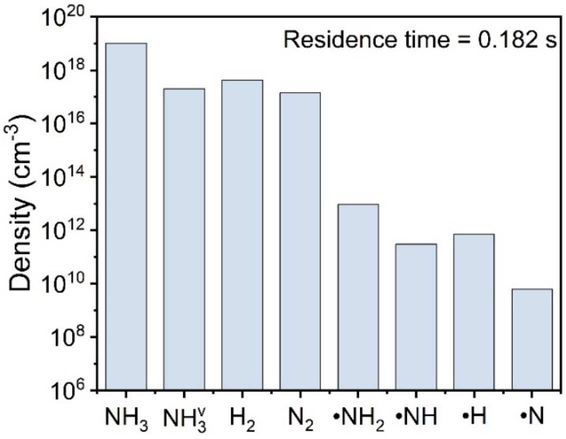

Figure S15. Estimated densities of plasma-induced vibrationally excited NH_3, and reactive radicals under plasma-only conditions at a residence time of 0.182 s based on the experimental reactor geometry and gas flow rate.[20]

5. Microkinetic Modeling

To account for the contributions of the zero-point energy (ZPE), temperature-dependent vibrational entropy ($S_{vib,\ i}$), and internal energy ($U_{vib,\ i}$) for initial state, transition state, and final state, the following equations are employed[17]:

$$ZPE = \frac{1}{2}\sum_i^N h\nu_i \tag{S15}$$

$$S_{vib,i} = k_B \sum_i^N \left[\frac{h\nu_i}{T\times exp\left(\frac{h\nu_i}{k_BT}-1\right)} + ln\left(\frac{1}{1-exp\left(-\frac{h\nu_i}{k_BT}\right)}\right)\right] \tag{S16}$$

$$U_{vib,i} = \sum_i^N \left[-k_BT \times ln\left(\frac{1}{1-exp\left(-\frac{h\nu_i}{k_BT}\right)}\right) + TS_{vib,i}\right] \tag{S17}$$

where h is the Planck constant, ν_i is the frequency of the ith vibrational mode, k_B is the Boltzmann constant, and T is the temperature. ΔU^{rxn} is the internal energy changes of the reaction between the initial and final states, ΔS^{rxn} is the entropy changes of the reaction between the initial and final states. ΔU^{TS} and ΔS^{TS} are the internal energy changes and entropy changes of the reaction between the transition and initial states.

The standard enthalpy change (ΔH^{rxn}) and the enthalpy change at the transition state (ΔH^{TS}) corrected with ZPE and temperature and Gibbs activation barrier of an elementary reaction step i is determined using the following equations:

$$\Delta H^{rxn} = \Delta E_{ZPE} + \Delta E_{DFT} + \Delta U^{rxn} \tag{S18}$$

$$\Delta H^{TS} = \Delta E_{ZPE}^{TS} + \Delta E_a^{TS} + \Delta U^{TS} \tag{S19}$$

here ΔE_{ZPE} corresponds to the zero-point energy (ZPE) correction, ΔE_{DFT} represents the electronic DFT reaction energy between the final and initial states. The terms, ΔE_{ZPE}^{TS}, ΔE_a^{TS}, and ΔU^{TS}, represent the ZPE correction, DFT-calculated activation energy, and internal energy changes between the transition state and the initial state, respectively.

In MKMs, reaction rate constants ($k_{f,\ i}$) for surface reactions are commonly described using the modified Arrhenius equation:

$$k_{f,i} = AT^{\beta} \times exp\left(-\frac{\Delta G^{TS}}{RT}\right) \tag{S20}$$

here A is the pre-exponential factor, T is temperature, β describes explicit temperature dependence (set as 1 according to Eyring-Polanyi equation), and R is the universal gas constant. For surface reactions, the pre-exponential factor A typically derives from TST as:

$$A = \frac{k_B}{h\sigma^{m-1}} \tag{S21}$$

with k_B is Boltzmann's constant, h is Planck's constant, σ is the surface site density, and m is the total number of surface species involved.

In this approach, the Gibbs activation energies (ΔG^{TS}) incorporate enthalpy and entropic contributions explicitly:

$$\Delta G^{TS} = \Delta H^{TS} - T\Delta S^{TS} \quad \text{(S22)}$$

Adsorption reactions are modelled using collision theory, characterized by a sticking coefficient (s), yielding adsorption rate constants (A^{ads}) as:

$$A^{ads} = \frac{s}{\sigma^m}\sqrt{\frac{RT}{2\pi M_i}} \quad \text{(S23)}$$

where M_i denotes the molecular weight of the adsorbing gas-phase species.

The Gibbs free energy (ΔG^{rxn}) of an elementary reaction step is determined using the following equation:

$$\Delta G^{rxn} = \Delta H^{rxn} - T\Delta S^{rxn} \quad \text{(S24)}$$

here ΔH^{rxn} is the standard enthalpy change and ΔS^{rxn} is the entropy change of the reaction between the initial and final states.

The equilibrium (K) and backward rate constant ($k_{b,\,i}$) can be estimated as follows[29, 30]:

$$K = exp\left(-\frac{\Delta G^{rxn}}{RT}\right) \quad \text{(S25)}$$

$$k_{b,i} = \frac{k_{f,i}}{K} \quad \text{(S26)}$$

5.1. Turnover Frequency (TOF) Calculation

The TOF,[31] defined as the number of ammonia molecules decomposed per active metal site per second (s^{-1}), is calculated from the catalytic performance data as:

$$\text{TOF} = \frac{(\text{NH}_3^{\text{in}} - \text{NH}_3^{\text{out}})}{\tau \times (\text{surface concentration of active sites}) \times (\text{catalyst area per unit volume})} \quad \text{(S27)}$$

Here, NH_3^{in} and NH_3^{out} represent ammonia concentrations at the reactor inlet and outlet, respectively, and τ is the residence time, defined as reactor length divided by linear velocity.

Table S5. Microkinetic model parameters.

Cluster	Feed flow rate (cm^3/min)	Area per volume (m^{-1})	Site density (mol/cm^2)	Conversion (%)
Co_3C(001)	35	3000	3.14×10^{-9}	1
Co_3N(001)	35	3000	3.94×10^{-9}	1
Co(0001)	35	3000	1.61×10^{-10}	1
Ru(0001)	35	3000	1.61×10^{-10}	1

5.2. Degree of Rate Control (DRC)

The DRC[32] for an elementary reaction step i, $X_{RC,\,i}$, is mathematically defined as:

$$X_{RC,\,i} = \left(\frac{\partial \ln(rate)}{\partial \ln(k_i)}\right)_{k_{j\neq i},K_i} \approx \frac{(\ln rate_{Perturbed} - \ln rate_{original})}{\delta \times \ln rate_{original}} \tag{S28}$$

where k_i is the rate constant for step i, δ is a small perturbation (5%) introduced to evaluate sensitivity, and $rate_{Perturbed}$ and $rate_{original}$ are rates after and before perturbation, respectively. To maintain thermodynamic consistency, the equilibrium constant K_i was kept constant by simultaneously varying forward and reverse rate constants.

5.3. Influence of the Reverse Reaction in Plasma-Assisted NH_3 Decomposition

Table S6. Elementary steps included in the microkinetic model to account for NH_3 synthesis pathways, incorporating radical-surface interactions and vibrationally assisted N_2 activation.

Reaction No	Reaction
1	$\bullet NH_2 + NH_3^* \rightleftharpoons NH_2^* + NH_3(g)$
2	$\bullet NH_2 + NH_2^* \rightleftharpoons NH^* + NH_3^*$
3	$\bullet NH_2 + NH_2^* \rightleftharpoons NH^* + NH_3(g)$
4	$\bullet NH_2 + NH^* \rightleftharpoons N^* + NH_3(g)$
5	$\bullet NH_2 + H^* \rightleftharpoons NH_3^*$
6	$\bullet H + NH_2^* \rightleftharpoons NH_3^*$
7	$N_2^{(v_0)} + 2^* \rightleftharpoons 2N^*$
8	$N_2^{(v_1)} + 2^* \rightleftharpoons 2N^*$
9	$N_2^{(v_2)} + 2^* \rightleftharpoons 2N^*$
10	$N_2^{(v_3)} + 2^* \rightleftharpoons 2N^*$
11	$N_2^{(v_4)} + 2^* \rightleftharpoons 2N^*$
12	$N_2^{(v_5)} + 2^* \rightleftharpoons 2N^*$
13	$N_2^{(v_6)} + 2^* \rightleftharpoons 2N^*$
14	$N_2^{(v_7)} + 2^* \rightleftharpoons 2N^*$
15	$N_2^{(v_8)} + 2^* \rightleftharpoons 2N^*$
16	$N_2^{(v_9)} + 2^* \rightleftharpoons 2N^*$
17	$N_2^{(v_{10})} + 2^* \rightleftharpoons 2N^*$

Our calculations (**Figure S16**) show that including the NH_3 formation pathway has a negligible effect on the decomposition rate on $Co_3C(001)$, and $Co_3N(001)$, Ru(0001), and Co(0001). The Plasma Catalysis-Original model excludes NH_3 formation steps, while Plasma Catalysis-Extended includes them; the two curves overlap across our investigated temperature range. Thus, we did not include the NH_3 synthesis steps in reporting the decomposition kinetics.

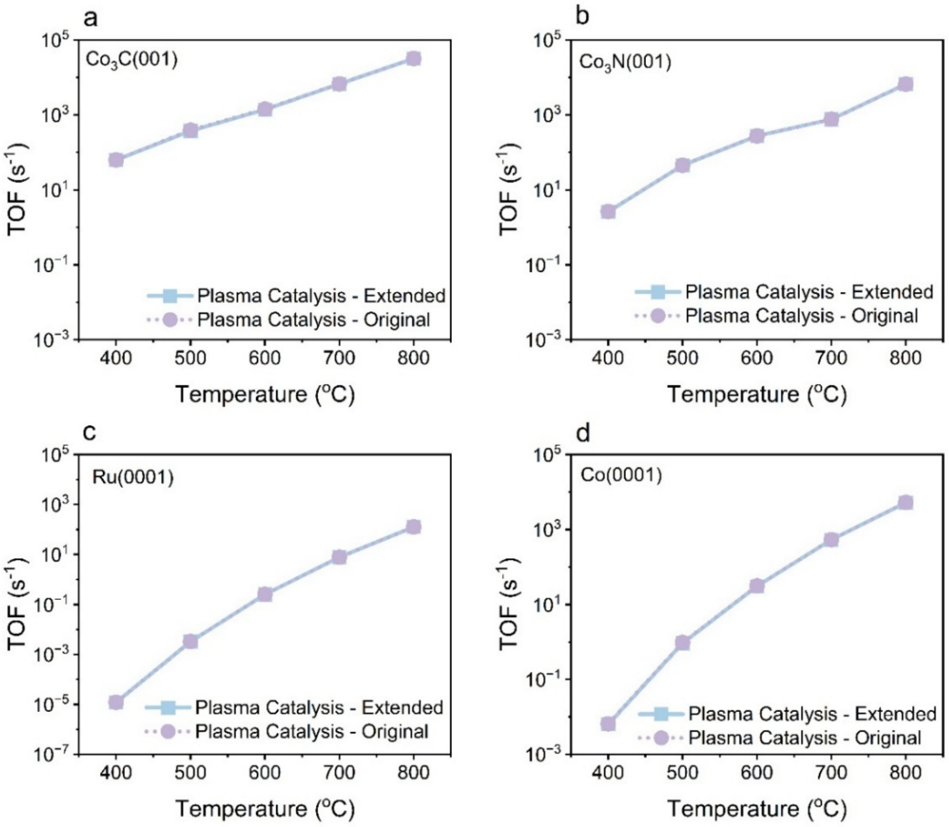

Figure S16. MKM-predicted TOFs for NH_3 decomposition over (a) $Co_3C(001)$, (b) $Co_3C(001)$, (c) Ru(0001), and (d) Co(0001) under plasma conditions, with and without including ammonia synthesis steps. Plasma Catalysis-Original excludes NH_3 synthesis steps, and Plasma Catalysis-Extended includes them. The nearly overlapping curves show that adding the NH_3 synthesis kinetics does not affect the decomposition kinetics over these catalysts.

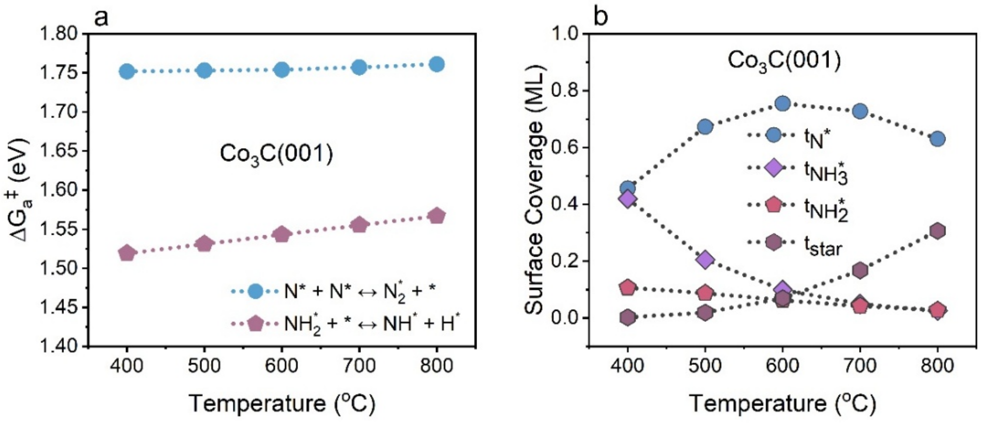

Figure S17. Temperature-dependent (a) Gibbs activation barriers of key elementary steps of N≡N bond formation and NH_2 dehydrogenation; and (b) surface coverages of abundant surface species of N^*, NH_3^*, NH_2^*, and surface empty sites (star) on $Co_3C(001)$ under thermal conditions.

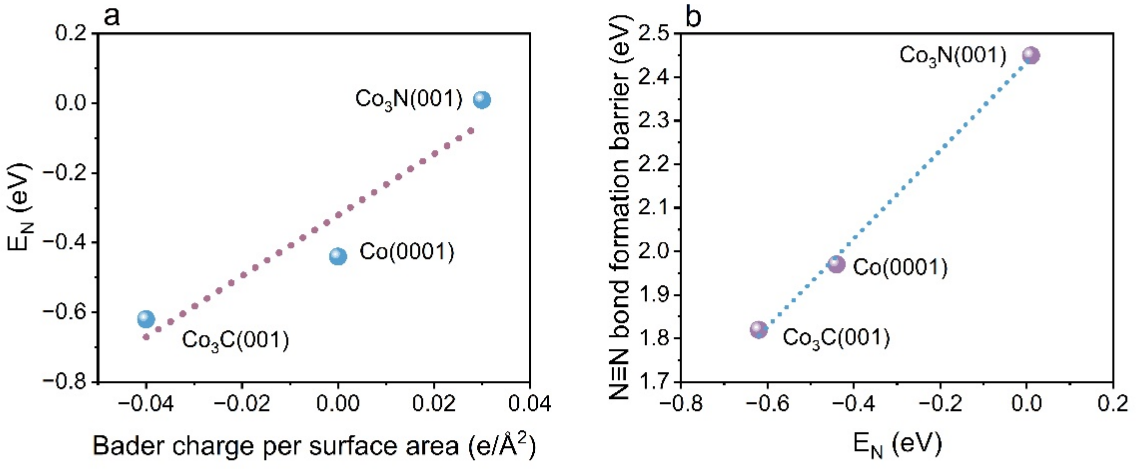

Figure S18. (a) Linear correlation between the top-layer surface charge per surface area of Co_3C, Co, and Co_3N catalysts and key intermediate N^* binding energy (E_N). (b) Linear relationship between E_N and the activation barrier for rate-limiting step of N≡N bond formation across $Co_3C(001)$, Co(0001), and $Co_3N(001)$ catalysts.

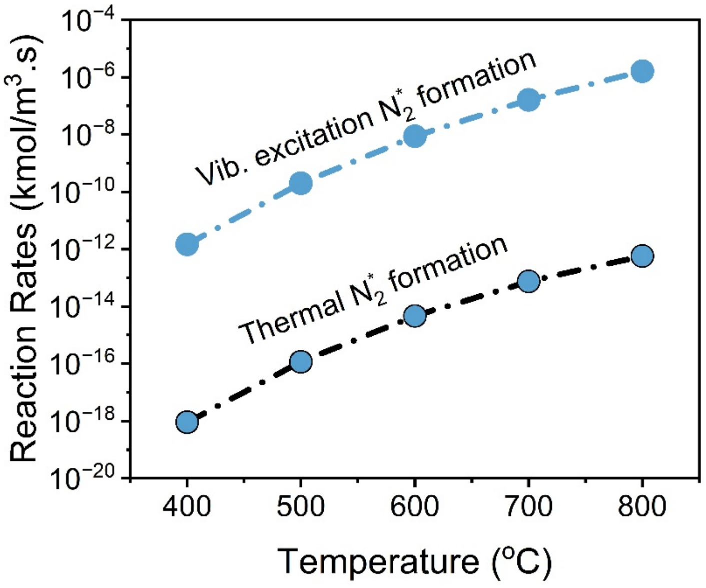

Figure S19. Temperature-dependent evolution of reaction rates for Co(0001).

5.4. Role of C and N under Plasma Conditions

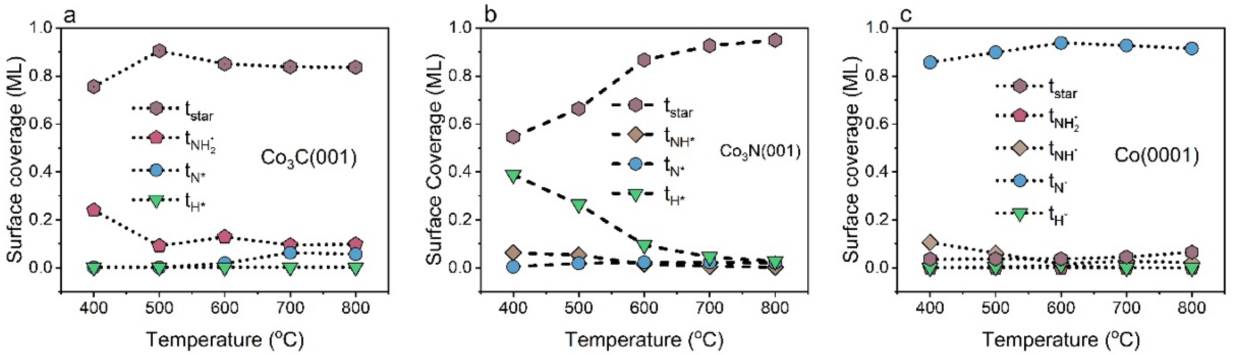

Figure S20. Temperature-dependent surface coverages of the most abundant intermediates under plasma conditions on (a) $Co_3C(001)$, (b) $Co_3N(001)$, and (c) Co(0001).

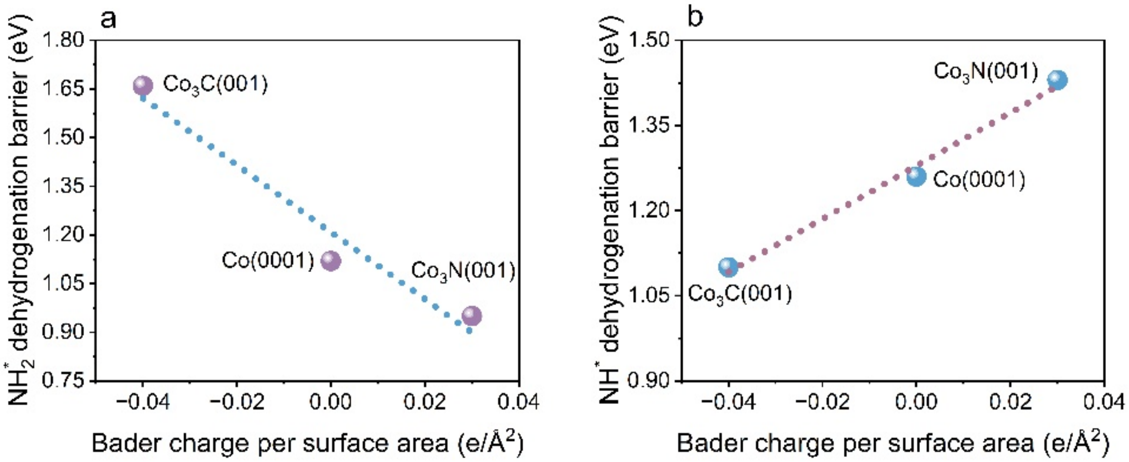

Figure S21. (a) Linear relationship between the top-layer surface charge per surface area of $Co_3C(001)$, Co(0001), and $Co_3N(001)$ and the activation barrier for NH_2^* dehydrogenation. (b) Linear relationship between the top-layer surface charge per surface area of $Co_3C(001)$, Co(0001), and $Co_3N(001)$ and the activation barrier for NH^* dehydrogenation.

References

1. Nagakura, S., Study of metallic carbides by electron diffraction part IV. Cobalt carbides. *Journal of the Physical Society of Japan* **1961,** *16* (6), 1213-1219.
2. Liu, B.; Li, W.; Xu, Y.; Lin, Q.; Jiang, F.; Liu, X., Insight into the intrinsic active site for selective production of light olefins in cobalt-catalyzed Fischer–Tropsch synthesis. *ACS Catalysis* **2019,** *9* (8), 7073-7089.
3. Balasubramanian, B.; Zhao, X.; Valloppilly, S. R.; Beniwal, S.; Skomski, R.; Sarella, A.; Jin, Y.; Li, X.; Xu, X.; Cao, H., Magnetism of new metastable cobalt-nitride compounds. *Nanoscale* **2018,** *10* (27), 13011-13021.
4. Widenmeyer, M.; Shlyk, L.; Becker, N.; Dronskowski, R.; Meissner, E.; Niewa, R., Synthesis of metastable Co_4N, Co_3N, Co_2N, and $CoO_{0.74}N_{0.24}$ from a single azide precursor and intermediates in $CoBr_2$ ammonolysis. *European Journal of Inorganic Chemistry* **2016,** *2016* (29), 4792-4801.
5. Sakurai, M.; Zhao, X.; Wang, C.-Z.; Ho, K.-M.; Chelikowsky, J. R., Influence of nitrogen dopants on the magnetization of Co_3N clusters. *Physical Review Materials* **2018,** *2* (2), 024401.
6. Jain, A.; Ong, S. P.; Hautier, G.; Chen, W.; Richards, W. D.; Dacek, S.; Cholia, S.; Gunter, D.; Skinner, D.; Ceder, G., Commentary: The Materials Project: A materials genome approach to accelerating materials innovation. *APL Materials* **2013,** *1* (1).
7. Marshall, P. V.; Alptekin, Z.; Thiel, S. D.; Smith, D.; Meng, Y.; Walsh, J. P., High-pressure synthesis of bulk cobalt cementite, Co_3C. *Chemistry of Materials* **2021,** *33* (24), 9601-9607.
8. Chen, Z.; Liu, L.; Chen, Q., One-pot template-free synthesis of urchin-like Co_2C/Co_3C hybrid nanoparticles. *Materials Letters* **2016,** *164*, 554-557.
9. Carroll, K. J.; Huba, Z. J.; Spurgeon, S. R.; Qian, M.; Khanna, S. N.; Hudgins, D. M.; Taheri, M. L.; Carpenter, E. E., Magnetic properties of Co_2C and Co_3C nanoparticles and their assemblies. *Applied Physics Letters* **2012,** *101* (1).
10. Harris, V.; Chen, Y.; Yang, A.; Yoon, S.; Chen, Z.; Geiler, A.; Gao, J.; Chinnasamy, C.; Lewis, L.; Vittoria, C., High coercivity cobalt carbide nanoparticles processed via polyol reaction: a new permanent magnet material. *Journal of Physics D: Applied Physics* **2010,** *43* (16), 165003.
11. Turgut, Z.; Lucas, M.; Leontsev, S.; Semiatin, S.; Horwath, J., Metastable Co_3C nanocrystalline powder produced via reactive ball milling: Synthesis and magnetic properties. *Journal of Alloys and Compounds* **2016,** *676*, 187-192.
12. Shen, X.; Ma, C.; Suo, H.; Zhang, T.; Yan, L.; Huang, L.; Zhou, J.; Wen, X.; Li, Y.; Yang, Y., Wet-chemistry approach for the synthesis of single phase ferromagnetic Co_3C nanoparticle. *Nano Select* **2021,** *2* (7), 1368-1371.
13. Kang, B. K.; Im, S. Y.; Lee, J.; Kwag, S. H.; Kwon, S. B.; Tiruneh, S.; Kim, M.-J.; Kim, J. H.; Yang, W. S.; Lim, B., In-situ formation of MOF derived mesoporous Co_3N/amorphous N-doped carbon nanocubes as an efficient electrocatalytic oxygen evolution reaction. *Nano Research* **2019,** *12* (7), 1605-1611.
14. Liu, Y.; Zhang, J.; Li, Y.; Qian, Q.; Li, Z.; Zhu, Y.; Zhang, G., Manipulating dehydrogenation kinetics through dual-doping Co_3N electrode enables highly efficient hydrazine oxidation assisting self-powered H_2 production. *Nature Communications* **2020,** *11* (1), 1853.

15. Zeinalipour-Yazdi, C. D.; Hargreaves, J. S.; Catlow, C. R. A., Low-T mechanisms of ammonia synthesis on Co_3Mo_3N. *The Journal of Physical Chemistry C* **2018,** *122* (11), 6078-6082.
16. Daisley, A.; Higham, M.; Catlow, C. R. A.; Hargreaves, J. S., Experimental and theoretical investigations on the anti-perovskite nitrides Co_3CuN, Ni_3CuN and Co_3MoN for ammonia synthesis. *Faraday Discussions* **2023,** *243*, 97-125.
17. Che, F.; Ha, S.; McEwen, J.-S., Elucidating the field influence on the energetics of the methane steam reforming reaction: A density functional theory study. *Applied Catalysis B: Environmental* **2016,** *195*, 77-89.
18. Li, H.; Reuter, K., Active-site computational screening: role of structural and compositional diversity for the electrochemical CO_2 reduction at Mo carbide catalysts. *ACS Catalysis* **2020,** *10* (20), 11814-11821.
19. Chase, M. W., NIST-JANAF Thermochemical Tables 4th ed. *Journal of Physical and Chemical Reffernce Data* **1998**, 1529-1564.
20. Ahmat Ibrahim, S.; Meng, S.; Milhans, C.; Barecka, M. H.; Liu, Y.; Li, Q.; Yang, J.; Sha, Y.; Yi, Y.; Che, F., Interpretable machine learning-guided plasma catalysis for hydrogen production. *Nature Chemical Engineering* **2025,** *2* (11), 699-710.
21. Wang, Z.; Qu, Y.; Shen, X.; Cai, Z., Ruthenium catalyst supported on Ba modified ZrO_2 for ammonia decomposition to CO_x-free hydrogen. *International Journal of Hydrogen Energy* **2019,** *44* (14), 7300-7307.
22. Yu, P.; Wu, H.; Guo, J.; Wang, P.; Chang, F.; Gao, W.; Zhang, W.; Liu, L.; Chen, P., Effect of BaNH, CaNH, Mg_3N_2 on the activity of Co in NH_3 decomposition catalysis. *Journal of Energy Chemistry* **2020,** *46*, 16-21.
23. Zhao, Z.; He, W.; Guo, B.; Yu, J.; Wang, Z.; Yu, H., A Comprehensive Review of Ammonia Decomposition for Hydrogen Production. *Energy & Fuels* **2025,** *39* (29), 13825-13847.
24. Meng, S.; Li, S.; Sun, S.; Bogaerts, A.; Liu, Y.; Yi, Y., NH_3 decomposition for H_2 production by thermal and plasma catalysis using bimetallic catalysts. *Chemical Engineering Science* **2024,** *283*, 119449.
25. Gao, Y.; Hu, E.; Yi, Y.; Yin, G.; Huang, Z., Plasma-assisted low temperature ammonia decomposition on 3d transition metal (Fe, Co and Ni) doped CeO_2 catalysts: Synergetic effect of morphology and co-doping. *Fuel Processing Technology* **2023,** *244*, 107695.
26. Zhu, X.; Liu, J.; Hu, X.; Zhou, Z.; Li, X.; Wang, W.; Wu, R.; Tu, X., Plasma-catalytic synthesis of ammonia over Ru-based catalysts: Insights into the support effect. *Journal of the Energy Institute* **2022,** *102*, 240-246.
27. Che, F.; Gray, J. T.; Ha, S.; Kruse, N.; Scott, S. L.; McEwen, J.-S., Elucidating the roles of electric fields in catalysis: a perspective. *ACS Catalysis* **2018,** *8* (6), 5153-5174.
28. Wan, M.; Yue, H.; Notarangelo, J.; Liu, H.; Che, F., Deep Learning-Assisted Investigation of Electric Field–Dipole Effects on Catalytic Ammonia Synthesis. *JACS Au* **2022,** *2* (6), 1338-1349.
29. Motagamwala, A. H.; Ball, M. R.; Dumesic, J. A., Microkinetic analysis and scaling relations for catalyst design. *Annual Review of Chemical and Biomolecular Engineering* **2018,** *9*, 413-450.
30. Mehta, P.; Barboun, P.; Herrera, F. A.; Kim, J.; Rumbach, P.; Go, D. B.; Hicks, J. C.; Schneider, W. F., Overcoming ammonia synthesis scaling relations with plasma-enabled catalysis. *Nature Catalysis* **2018,** *1* (4), 269-275.

31. Singh, P.; Li, Q.; Liu, Y.; Che, F., Multiscale Simulation Guided Electric Field-Enhanced Ammonia Catalytic Cracking. *ACS Catalysis* **2025,** *15* (10), 7690-7699.
32. Wolcott, C. A.; Medford, A. J.; Studt, F.; Campbell, C. T., Degree of rate control approach to computational catalyst screening. *Journal of Catalysis* **2015,** *330*, 197-207.
